# Genetic and chemical markers for authentication of three *Artemisia* species: *A*. *capillaris*, *A*. *gmelinii*, and *A*. *fukudo*

**DOI:** 10.1371/journal.pone.0264576

**Published:** 2022-03-10

**Authors:** Yun Sun Lee, Sunmin Woo, Jin-Kyung Kim, Jee Young Park, Nur Kholilatul Izzah, Hyun-Seung Park, Jung Hwa Kang, Taek Joo Lee, Sang Hyun Sung, Kyo Bin Kang, Tae-Jin Yang

**Affiliations:** 1 Department of Plant Science, Plant Genomics and Breeding Institute, Research Institute of Agriculture and Life Sciences, College of Agriculture and Life Sciences, Seoul National University, Seoul, Republic of Korea; 2 College of Pharmacy and Research Institute of Pharmaceutical Sciences, Seoul National University, Seoul, Republic of Korea; 3 Hantaek Botanical Garden, Yongin, Republic of Korea; 4 Research Institute of Pharmaceutical Sciences, College of Pharmacy, Sookmyung Women’s University, Seoul, Republic of Korea; Institute for Biological Research, University of Belgrade, SERBIA

## Abstract

The genus *Artemisia* is an important source of medicines in both traditional and modern pharmaceutics, particularly in East Asia. Despite the great benefits of herbal medicine, quality assessment methods for these medicinal herbs are lacking. The young leaves from *Artemisia* species are generally used, and most of the species have similar morphology, which often leads to adulteration and misuse. This study assembled five complete chloroplast genomes of three *Artemisia* species, two accessions of *A*. *gmelinii* and *A*. *capillaris*, and one *A*. *fukudo*. Through comparative analysis, we revealed genomic variations and phylogenetic relationships between these species and developed seven InDel-based barcode markers which discriminated the tested species from each other. Additionally, we analyzed specialized metabolites from the species using LC-MS and suggested chemical markers for the identification and authentication of these herbs. We expect that this integrated and complementary authentication method would aid in reducing the misuse of *Artemisia* species.

## Introduction

The genus *Artemisia* (Asteraceae), mainly native to Central Asia, consists of more than 500 taxa at a specific or subspecific level [1,2]. In East Asian traditional medicine, *Artemisia* species have been widely used to treat jaundice, liver diseases, and renal failure [3]. Specialized metabolites biosynthesized by *Artemisia* species, especially phenolic compounds, exhibit potent bioactivities, including antibiotic, antioxidant, hepatoprotective, and antifibrotic effects [4]. The Nobel Prize awardee Youyou Tu’s discovery of artemisinin from *A*. *annua* highlights the potential of specialized metabolites as a drug discovery source and the ethnopharmacological value of these medicinal plants [5].

The large genetic diversity of *Artemisia* results in a high variety of morphologies and phytochemical contents. Based on the infrageneric classification inferred from the nuclear ribosomal internal transcribed spacer (ITS), *Artemisia* species are categorized into five subgenera: *Artemisia*, *Absinthium*, *Dracunculus*, *Seriphidium*, and *Tridentata* [6]. The subgenus *Artemisia* is the most heterogeneous in morphological, chemical, ecological, and karyological characteristics [1]. *A*. *gmelinii* Webb. ex Stechm., belonging to the subgenera *Artemisia*, and *A*. *capillaris* Thunb., belonging to the subgenus *Dracunculus*, are consumed as medicinal herbs, especially the young leaves. However, the morphology of the young leaves is similar. This complicates species identification and leads to adulteration or misuse in herbal markets. Thus, a validated method to authenticate *Artemisia* species needs to be developed [3].

DNA barcoding has been suggested as a reliable tool for identifying plant species used as food and herbal medicines. The ITS and chloroplast regions, such as intergenic regions between *trnH*—*psbA*, *psbK*—*psbI*, and *atpF*—*atpH*, and genic regions including *rpoB*, *rpoC1*, *rbcL*, and *matK*, are used to classify intergenic or intragenic species and to understand the taxonomic relationship between species [7]. DNA barcoding markers for the *Artemisia* species were developed using the ITS and chloroplast intergenic regions between *trnH—psbA* and *trnL—trnF*. A sequence characterized amplified regions (SCAR) marker was developed to discriminate *A*. *princeps* and *A*. *argyi* from *A*. *capillaris* and *A*. *iwayomogi* [8], *and the trnL*-*F* region of the chloroplast genome was used to discriminate *A*. *apiacea*, *A*. *keiskeana*, and *A*. *sieversiana* from 18 other *Artemisia* species [3]. High-resolution melting analysis based on ITS2 sequences successfully discriminated *A*. *argyi*, *A*. *annua*, *A*. *lavandulaefolia*, *A*. *indica*, and *A*. *atrovirens* [9]. ITS2 and *psbA—trnH* were applied to identify *A*. *argyi* from 15 closely related species and counterfeits [10], and ITS, ITS2, and *psbA—trnH* were able to identify *A*. *annua*, *A*. *absinthium*, *A*. *rupestris*, *A*. *tonurnefortiana*, *A*. *austriaca*, *A*. *dracunculus*, *A*. *vulgaris*, and *A*. *macrocephala* [11]. However, universal DNA barcoding markers developed from plastids, widely used for the discrimination of plant species, may not be applicable for some species because of the transfer of ancestral plastid genomes into the mitochondrial genome [12]. This suggests the need to develop genetic markers specific to a given species. Limiting intraspecific variation by collecting multiple samples of a species from different geographic origins is also needed to understand the natural intraspecific diversity of wild-crafted herbal materials and prevent potential false-positive or false-negative authentication errors [12].

Another typical authentication method for medicinal plants is the chemical profiling of specialized metabolites. Chemical profiling is not sufficiently accurate for taxonomic identification because the metabolites can differ due to many non-genetic factors, such as climate, time of harvest, and symbiosis. However, chemical profiling has advantages, especially when DNA extraction is impossible. For example, authentication is required for crude extracts or manufactured goods. Previously, we showed that HPLC-UV-based chemical analysis could be used to discriminate between *A*. *princeps* and *A*. *capillaris* [13]. It was reported that HPLC-UV [14], GC-MS [15], and LC-MS [16] are powerful tools for chemical profiling of and discrimination between *Artemisia* species. This study suggests chloroplast genome-based DNA barcodes for three *Artemisia* species (i.e., *A*. *capillaris*, *A*. *gmelinii*, and *A*. *fukudo* Makino) for which DNA barcodes were not suggested previously. A previous study suggested that *psbA–trnH* is highly variable region in the *Artemisia* species [17]; however, as the region did not show high level of variations between *A*. *gmelinii*, *A*. *capillaris*, and *A*. *fukudo*, we developed DNA barcoding markers specific to these species based on the other regions. Additionally, we analyzed specialized metabolites of these three species using LC-MS/MS to provide ground knowledge on the development of chemical markers for authentication.

## Materials and methods

### Plant materials

Samples of *A*. *gmelinii*, *A*. *capillaris*, and *A*. *fukudo* were collected from different regions in Korea and maintained at the Hantaek Botanical Garden (Yongin, Korea) or Department of Herbal Crop Research, Rural Development Administration (Eumseong, Korea). *A*. *gmelinii* and *A*. *capillaris* were biologically duplicated by collecting individuals from different geographical locations. The biological duplicates were named *A*. *gmelinii*-A, *A*. *gmelinii*-B, *A*. *capillaris*-A, and *A*. *capillaris*-B. Detailed information on the collection site and the location of each sample is described in S1 Table. The plants were grown in a greenhouse, with the light, temperature, and humidity maintained at similar levels across the greenhouse.

### Chemicals and reagents

HPLC-grade water, MeOH, and MeCN were purchased from Avantor Performance Materials Inc. (Center Valley, PA, USA). Formic acid and leucine enkephalin were purchased from Sigma–Aldrich (St. Louis, MO, USA). Reference standards for the identification of LC-MS/MS peaks were purchased as follows: neochlorogenic acid (**2**) from Interpharm (Koyang, Korea), scopoletin (**12**) from Sigma Aldrich, chlorogenic acid (**6**), hyperoside (**16**), luteolin-7-*O*-glucoside (**18**), and isorhamnetin-3-*O*-*β*-d-glucoside (**24**) from Chromadex (Irvine, CA, USA).

### Chloroplast genome sequencing and annotation

Total genomic DNA was extracted from the leaf tissue of each *Artemisia* species using a modified cetyltrimethylammonium bromide method [18]. Our previous studies described the methods for chloroplast genome assembly of *A*. *gmelinii*, *A*. *capillaris*, and *A*. *fukudo* [19,20]. The three *Artemisia* species were sequenced using the Illumina MiSeq platform (Illumina, San Diego, CA, USA). High-quality paired-end reads of 1.2–2.1 Gb were generated and separately assembled using CLC genome assembler 4.6 (CLC Inc., Aarhus, Denmark). All contigs representing chloroplast sequences were retrieved, ordered, and combined into a single sequence based on the chloroplast genome of *A*. *frigida* (JX293720) [21]. Sequences can be found in GenBank under the following accession numbers: KU736962 for *A*. *gmelinii*-A, KY073390 for *A*. *gmelinii*-B, KU736963 for *A*. *capillaris*-A, KY073391 for *A*. *capillaris*-B, and KU360270 for *A*. *fukudo*.

Complete chloroplast genomes of the three *Artemisia* species were annotated using DOGMA [22] and manually corrected by comparison with chloroplast genomes deposited in GenBank. Chloroplast genome maps of the three *Artemisia* species were drawn using OGDRAW [23]. The genomic differences among the three *Artemisia* species were analyzed using Mvista [24]. The repeat sequences, including forward, palindrome, reverse, and complement, were identified using the REPuter software [25] with the following criteria: cutoff n ≥30 bp and a sequence identity of ≥90%.

### Phylogenetic analysis

A phylogenetic tree was constructed using 64 protein coding sequences extracted from 18 species belonging to the Asteraceae family including *A*. *fukudo* and 2 sequences each from *A*. *gmelinii* and *A*. *capillaris*. The accession numbers of the chloroplast genomes used for phylogenetic analysis are listed in S2 Table. All coding sequences were aligned using MAFFT [26]. A phylogenetic tree was constructed using the neighbor-joining method with 1000 bootstrap values, as implemented in MEGA 6.0 [27].

### Development of InDel barcode markers and blind test analysis

InDel barcode markers were developed based on polymorphic regions identified in the aligned chloroplast genome sequences of *A*. *gmelinii*, *A*. *capillaris*, and *A*. *fukudo*. Interspecific polymorphic targets without intraspecific diversity among three species were selected as candidate regions for marker development. Primer sets were designed using Primer 3 version 0.4.0 [28], and the primer set information can be found in S3 Table. PCR was performed in a final volume of 25 μL, consisting of 20 ng DNA, 2 units of Taq polymerase (Vivagen, Seongnam, Korea), 2.5 mM dNTPs, and 20 pmol of each primer. The PCR conditions were as follows: 5 min at 94°C; 35 cycles of 30 s at 94°C, 30 s at 54°C, and 20 s at 72°C; and 7 min at 72°C as the final extension. PCR amplicons were analyzed on a 3% agarose gel and visualized under UV trans-illuminator using a gel documentation system.

Five InDel-based barcode markers specific to *A*. *gmelinii* and *A*. *capillaris* were used in a blind test on 20 dried commercial *Artemisia* species provided by the Ministry of Food and Drug Safety of Korea. The samples were labeled as follows: seven as *A*. *gmelinii*, six as *A*. *capillaris*, six as *A*. *apiacea*, and one as *A*. *annua*. The amplified PCR products were genotyped by capillary electrophoresis using a fragment analyzer (Advanced Analytical Technologies Inc., USA), according to the manufacturer’s protocol. The genotyping results were obtained by analysis using the PROSize 2.0 software (Agilent Technologies Inc., Santa Clara, CA, USA).

### LC-MS/MS-based specialized metabolite profiling

Accurately weighed powdered plant samples (100.0 mg) were extracted with 1.0 mL MeOH/H_2_O (5:5, v/v) and sonicated at room temperature for 15 min. The extracts were centrifuged at 15,000 × *g* for 10 min, and the supernatant (0.5 mL) was collected. Chicoric acid (Interpharm) was added as an internal standard (IS) at a 0.1 mg/mL final concentration. The samples were filtered through 0.20 μm Minisart RC15 filters (Sartorius Stedim Biotech, Göttingen, Germany). Each sample was analyzed in triplicate.

The samples were analyzed using a Waters Acquity UPLC system (Waters Co., Milford, MA, USA) coupled with a Waters Xevo G2 Q/TOF mass spectrometer (Waters MS Technologies, Manchester, UK) equipped with an electrospray ionization interface (ESI). Analytes were separated on a Waters Acquity UPLC HSS C_18_ column (1.8 μm, 2.1 × 100 mm) eluted with a mixture of 0.1% formic acid in water (solvent A) and acetonitrile containing 0.1% formic acid (solvent B) with the following gradient: 5% B, 0 min; 10% B, 5 min; 10% B, 6 min; 16% B, 7 min; 20% B, 13 min; 50% B, 18 min; 100% B, 20 min; 100% B, 23 min, 5% B, 23.1 min; 5% B, 26 min. The flow rate was 0.3 mL/min, and the injection volume was 1.0 μL. MS/MS data were acquired in the data-independent acquisition (MS^E^) mode. The detailed parameters of the MS setting are described in the S1 File. The order of data acquisition was randomized. A quality control (QC) sample (a mixture of every analyzed sample) was prepared and used to precondition the UHPLC column with five continuous injections. The QC sample was also injected after every five samples to monitor the precision of the analysis. Raw LC-MS/MS data were deposited in MassIVE (https://massive.ucsd.edu) under the accession number MSV000087782.

LC-MS data were processed to extract MS features and peak area-based intensities using MZmine 2.53 software [29]. The detailed parameters are described in S4 Table. The intensity of each ion was normalized to the IS intensity. Principal component analysis (PCA) was performed using the Python package *pca* (https://github.com/erdogant/pca). One-way ANOVA was performed using GraphPad Prism (version 7.0; GraphPad Software, Inc., La Jolla, CA, USA).

## Results and discussion

### Development of chloroplast genome in the three *Artemisia* species

**Fig 1:**
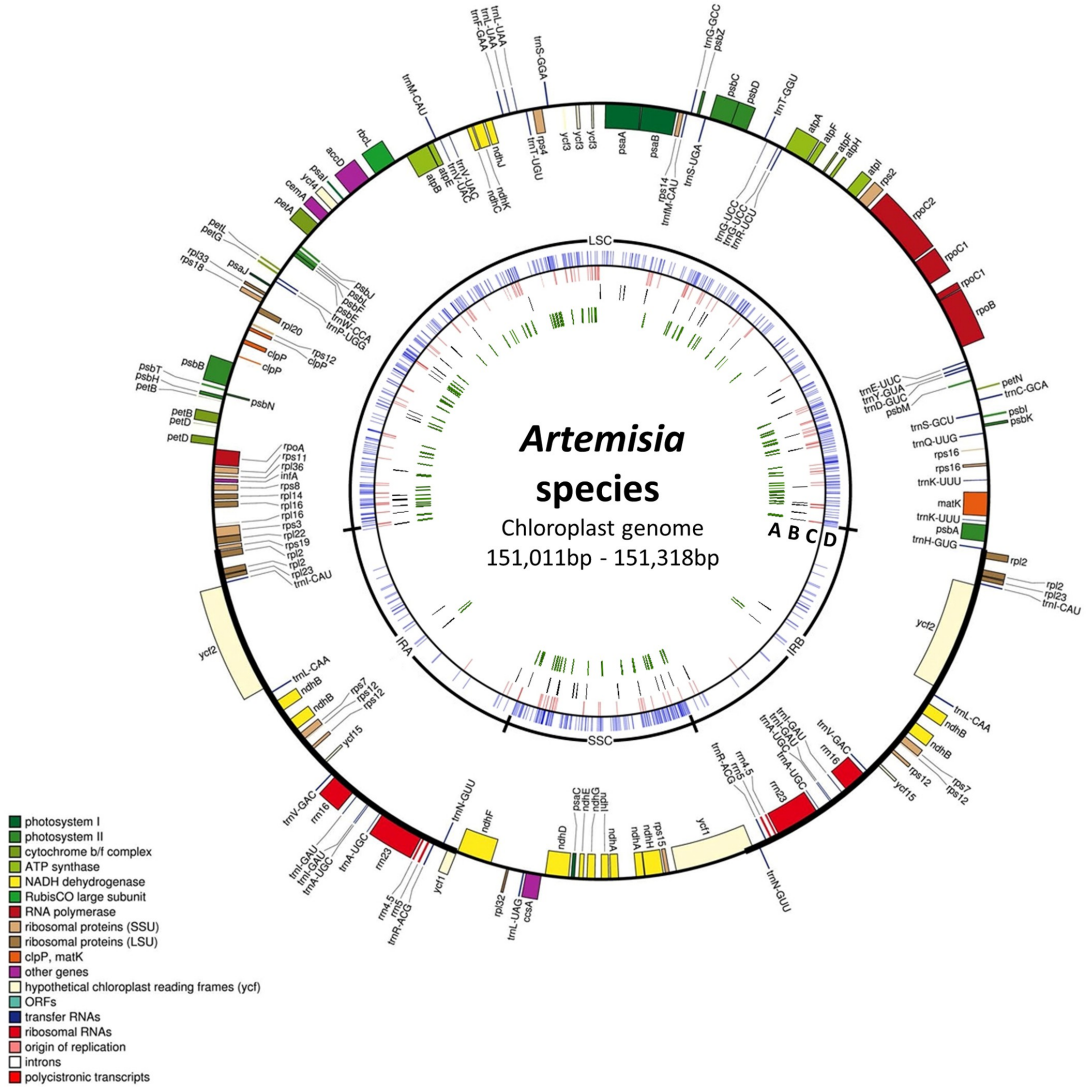
Chloroplast genome maps of *A*. *gmelinii*, *A*. *capillaris*, and *A*. *fukudo*. Genes represented on the outside of the outer circle are transcribed clockwise, and those shown on the inside of the outer circle are transcribed counterclockwise. Genes belonging to the same functional group were indicated in the same color. The inner tracks represent inter- and intraspecific variations. Track A and B are intraspecific variations of *A*. *gmelinii* and *A*. *capililaris*, respectively. Track C and D indicate total SNPs and InDels among five *Artemisia* chloroplast genomes, respectively.

**Fig 2:**
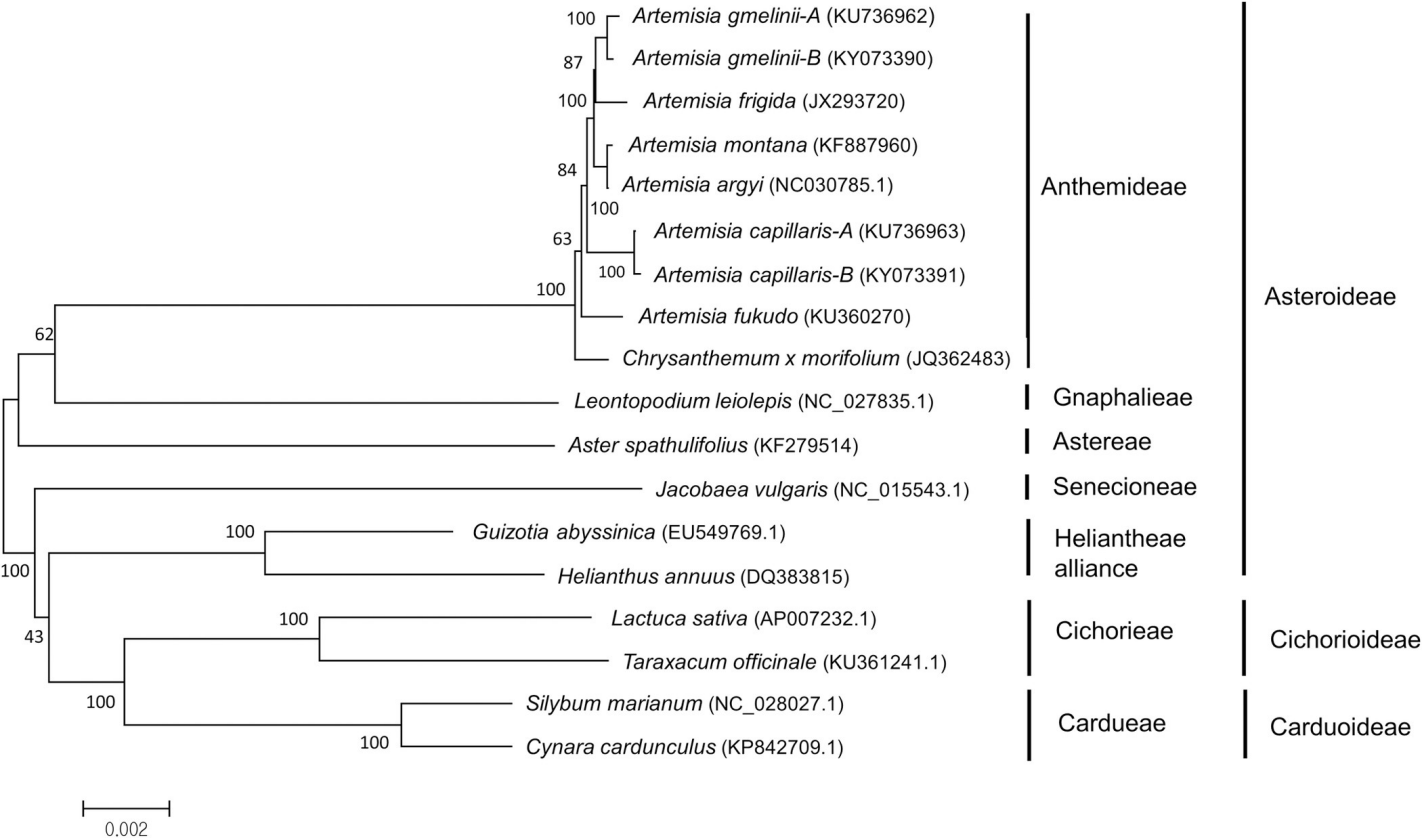
A phylogenetic tree of 18 species belonging to the Asteraceae family. A total of 64 coding regions in the chloroplast genome were used to analyze phylogenetic relationships among the species. The phylogenetic tree was constructed by the neighbor-joining method with 1000 bootstrap values. Numbers on each node were bootstrap values. The accession number of the chloroplast genomes used for the phylogenetic tree are in parenthesis and S2 Table.

Previous studies assembled the chloroplast genomes of three *Artemisia* species: *A*. *fukudo* [30], *A*. *gmelinii*, and *A*. *capillaris* [31]. In this study, we completed chloroplast genome sequences from different individuals of *A*. *gmelinii* (*A*. *gmelinii-*B*)* and *A*. *capillaris* (*A*. *capillaris-*B) and compared them with previously characterized sequences (*A*. *gmelinii-*A and *A*. *capillaris-*A) (Fig 1). The completed chloroplast genomes had a typical quadripartite structure consisting of a long single-copy section (LSC), inverted repeat (IR), and short single-copy section (SSC) (Table 1). The genome sizes were similar among the three species, ranging from 151,011 bp to 151,318 bp. Variation was detected within the same species: 151,318 bp for *A*. *gmelinii*-A and 151,050 bp for *A*. *gmelinii*-B, and 151,056 bp for *A*. *capillaris*-A and 151,020 bp for *A*. *capillaris*-B. The genome size of *A*. *fukudo* was 151,011 bp. The variations in genome size between intra- and inter-species were mainly due to the LSC region, as the lengths varied from 82,751 to 83,061 bp. Significant length differences of 271bp in the LSC region were observed between *A*. *gmelinii-*A and *A*. *gmelinii-*B. This difference came from 43 intraspecific InDels in the LSC region; most were in intergenic regions, except for three exonic ones. The average GC content of the chloroplast genomes was almost identical (37.4–37.5%) in the three species. We constructed a phylogenetic tree with 64 protein-coding sequences from the chloroplast genomes of 18 species, including seven tribes and three subfamilies of the Asteraceae family (Fig 2). For *Artemisia*, three additional species (i.e., *A*. *frigida*, *A*. *montana*, and *A*. *argyi*), were incorporated, as these chloroplast genome sequences are publicly available in NCBI. Overall, the phylogenetic tree was well-clustered by tribes and subfamilies. The six *Artemisia* species were grouped in the same clade along with *Chrysanthemum × morifolium* within the tribe Anthemideae, which is consistent with the phylogenetic analysis based on the protein-coding regions in chloroplasts [32]. Two individuals of *A*. *gmelinii* and *A*. *capillaris* collected from different locations were placed in the same clade, indicating that the individuals were most likely the same species.

**Table 1:** Summary of the NGS data and chloroplast genomes of *A*. *gmelinii*, *A*. *capillaris*, and *A*. *fukudo*.

sample	number of raw reads	total read length (bp)	mapped read number	chloroplast genome coverage (×)	chloroplast genome length (bp)	long single copy (LSC)	inverted repeat (IR)	short single copy (SSC)
*A*. *gmelinii*-A	7,295,868	2,191,126,001	187,330	300.24	151,318	83,061	24,961	18,335
*A*. *gmelinii*-B	8,762,916	2,626,280,574	52,077	78.01	151,050	82,790	24,961	18,338
*A*. *capillaris*-A	5,360,722	1,610,083,995	121,704	193.74	151,056	82,821	24,963	18,309
*A*. *capillaris*-B	9,532,322	2,859,007,916	143,982	217.70	151,020	82,786	24,963	18,302
*A*. *fukudo*	4,314,036	1,289,617,713	61,476	93.82	151,011	82,751	24,956	18,348

### Comparative analysis on chloroplast genomes for barcode marker development

**Fig 3:**
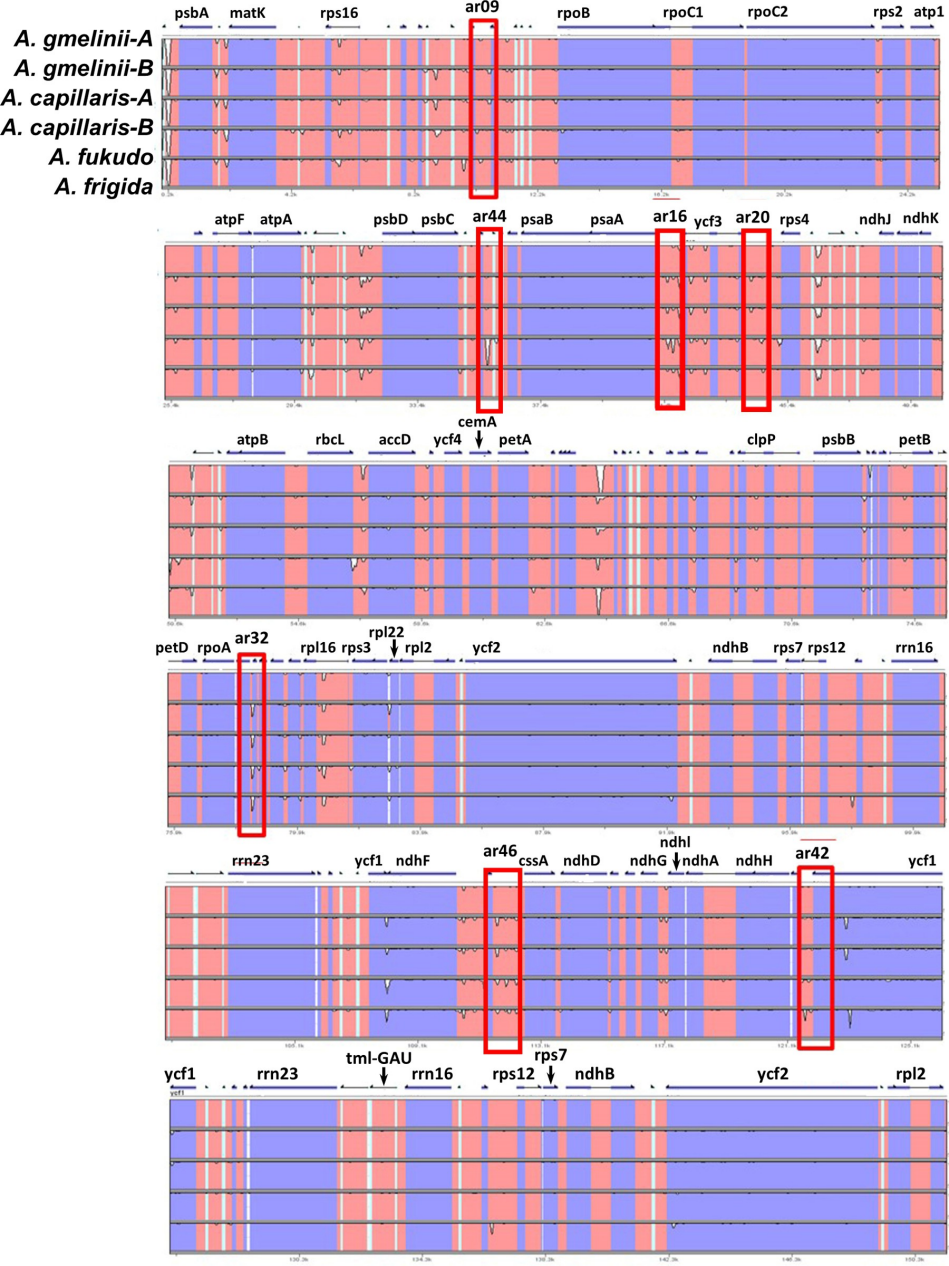
Chloroplast sequence variation between *A*. *gmelinii*, *A*. *capillaris*, *A*. *fukudo*, and *A*. *frigida*. The molecular markers were developed from the highly variable regions indicated by a red rectangle. The names of markers are shown above the red boxes.

For a deeper investigation of variations between the *Artemisia* chloroplast genomes, three *Artemisia* species were compared to previously reported *A*. *frigida* [21], with annotation of *A*. *gmelinii*-A as a reference. While more sequence variations were found in non-coding regions than in coding regions in all species, the 47 genes had at least one genic SNP. Seven genes, i.e., *matK*, *rpoB*, *rpoC2*, *accD*, *ndhF*, *ycf2*, and *ycf1*, were highly divergent among the three *Artemisia* species (Fig 3). Intraspecific variations were found in fifteen genes of *A*. *gmelinii* and twelve of *A*. *capillaris* (Figs 1 and 3). The coding regions of *A*. *fukudo* were less conserved than those of the other *Artemisia* species. As in other plant species [16,21,33], *ycf1* and *accD* were highly varied regions, suggesting these particular genes are prone to natural variations in plant chloroplasts. Regarding SNPs and InDels, more intraspecific differences were observed in *A*. *gmelinii* than in *A*. *capillaris*, with 107 and 30 SNPs, respectively. The number of InDels was 43 and 10 for *A*. *gmelinii* and *A*. *capillaris*, respectively (Table 2). Individuals of the same species might have undergone independent mutation events in different growing regions.

**Table 2:** The number of SNPs and InDels found in chloroplast genomes of three *Artemisia* species.

Samples	*A*. *gmelinii*-A	*A*. *gmelinii*-B	*A*. *capillaris*-A	*A*. *capillaris*-B	*A*. *fukudo*
*A*. *gmelinii*-A		43	102	104	119
*A*. *gmelinii*-B	107 (0.07)		91	91	107
*A*. *capillaris*-A	448 (0.30)	456 (0.30)		10	114
*A*. *capillaris*-B	458 (0.30)	462 (0.31)	30 (0.02)		113
*A*. *fukudo*	453 (0.30)	463 (0.31)	544 (0.36)	548 (0.36)	

The upper triangle shows the number of InDels, and the lower triangle indicates the total nucleotide substitutions. The percentage ratios of nucleotide variations to chloroplast genome sequences are given in brackets.

**Fig 4:**
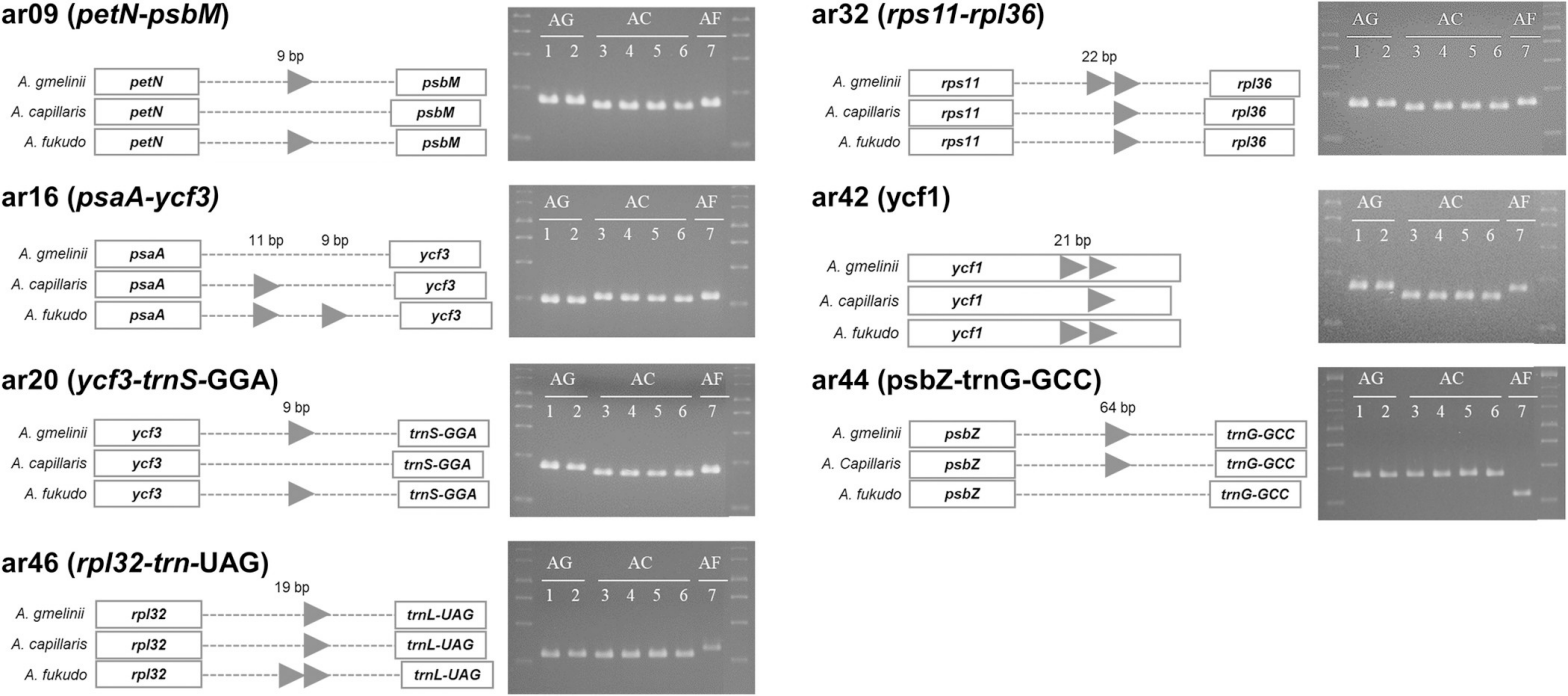
Discrimination success for *A*. *gmelinii*, *A*. *capillaris*, and *A*. *fukudo* using InDel-based barcode markers. Rectangles and dashed lines indicate the genes and intergenic regions, respectively. Arrows represent the inserted sequences within the intergenic regions. The amplified DNA fragments using the InDel-based barcode markers were separated on an agarose gel as shown on the right side (AG, *A*. *gmelinii*; AC, *A*. *capillaris*; AF, *A*. *fukudo*). The detailed sequence variations between the species are presented in S1 Fig.

Comparative analyses of the chloroplast genomes revealed the possibility of developing InDel-based barcode markers, providing useful guidance for identifying *Artemisia* species. We designed seven markers ar09, ar16, ar20, ar32, ar42, ar44, and ar46 based on the polymorphic regions of *petN*-*psbM*, *psaA*-*ycf3*, *ycf3*-*trnS*(GGA), *rps11*-*rpl36*, *ycf1*, *psbZ-trnG*(GCC), and *rpl32-trn*(UAG) (Figs 4 and S1). Five markers, ar09, ar16, ar20, ar32, and ar42, were used to distinguish between *A*. *gmelinii* and *A*. *capillaris*. Two markers, ar44 and ar46, were specifically identified in *A*. *fukudo*. PCR analysis showed that the developed markers produced PCR amplicons of different sizes, regardless of intraspecific variation in *A*. *capillaris* and *A*. *gmelinii*, with successful discrimination among the three species (Fig 4).

We then tested whether our barcode markers could be applied to real-world *Artemisia* products. Twenty dried herbs labeled *A*. *gmelinii*, *A*. *capillaris*, *A*. *annua*, and *A*. *apiacea*, were collected from the market and tested. Five markers, ar09, ar16, ar20, ar32, and ar42, successfully distinguished *A*. *gmelinii* and *A*. *capillaris* (S2 Fig). We found that *A*. *annua* and *A*. *apiacea* exhibited the same band pattern as *A*. *gmelinii* and *A*. *capillaris* on the gel, except for the result from the ar16 marker. This suggests that the two herbs might outcross, and multiple barcode markers are necessary for accurate validation [12]. This result demonstrated that InDel-based barcode markers could be utilized in the identification and authentication of *Artemisia* species.

### LC-MS/MS-based analysis on specialized metabolome

**Fig 5:**
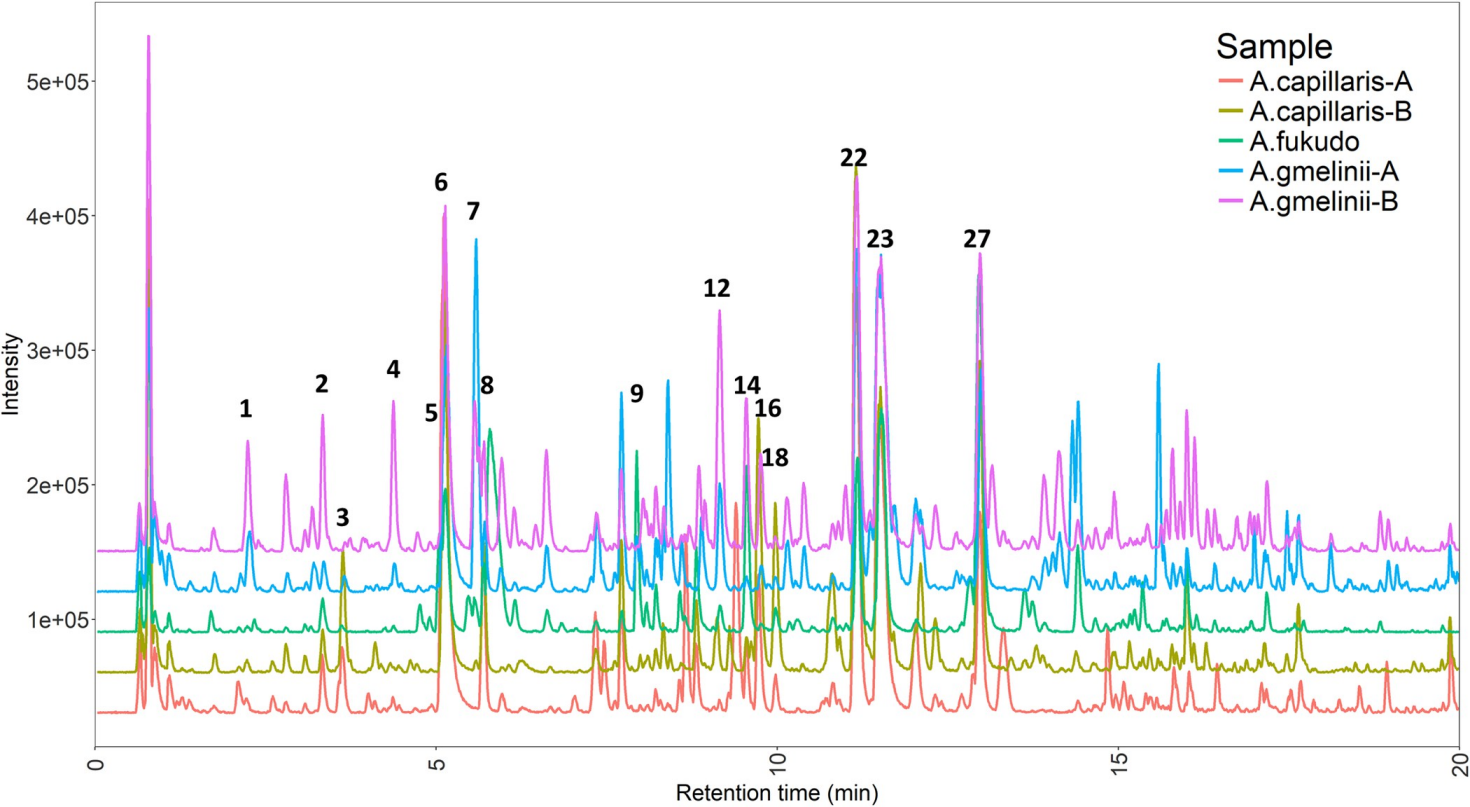
Representative LC-MS base peak ion (BPI) chromatograms of the *Artemisia* extracts. Tentatively annotated chromatographic peaks are annotated with peak numbers. Gaps between the chromatograms were added to help visualize the differences, so the y-axis values do not equal the absolute intensities.

Although chemical markers are useful for authentication of herbal products from which DNA extraction is impossible, it is difficult to develop valid chemical markers for identification, because the contents of specialized metabolites are heavily influenced by environmental factors. Nevertheless, we analyzed the *Artemisia* samples using LC-MS/MS, as a preliminary effort to gather background knowledge on their specialized metabolome. To minimize environmental effects on the accumulation or induction of specialized metabolites, all the plants were grown in a same greenhouse. As shown in Fig 5, the chemical profiles of the *Artemisia* extracts showed qualitative and quantitative interspecific variety. Twenty-eight peaks were putatively identified in the different Metabolomics Standards Initiative (MSI) confidence levels [34] (Table 3). Six peaks (**2**, **6**, **12**, **16**, **18**, and **25**) were identified at MSI level 1 using commercial standards, seventeen peaks were annotated at MSI level 2 by MS/MS spectral matching to the GNPS spectral library [35] (S3 Fig), and five peaks were annotated at level 3 by comparison of relative retention time and MS data with previous studies [16,36]. Most of the identified metabolites were polyphenols such as phenylpropanoids, flavonoids, and coumarins.

**Table 3:** Chromatographic and spectrometric data of the 28 tentatively identified metabolites from *Artemisia* species.

peak number	identification	t_R_ (min)	calculated *m/z* [M−H]^−^	observed *m/z* [M−H]^−^	mass error (ppm)	molecular formula	MSI identification level
**1**	dihydroxybenzoic acid hexoside	2.2	315.1716	315.0718	0.6	C_13_H_16_O_9_	3
**2**	neochlorogenic acid	3.3	353.0873	353.0873	0.0	C_16_H_18_O_9_	1
**3**	dihydroxybenzoic acid pentoside	3.7	285.0610	285.0605	-1.8	C_12_H_14_O_8_	2
**4**	1,3-*O*-dicaffeoylquinic acid	4.4	515.1401	515.1401	0.0	C_22_H_28_O_14_	3
**5**	caffeic acid	5.1	179.0344	179.0338	-3.4	C_9_H_8_O_4_	2
**6**	3-*O*-caffeoylquinic acid	5.1	353.0873	353.0871	-0.6	C_16_H_18_O_9_	1
**7**	*p*-coumaric acid hexoside	5.6	325.0923	325.0919	-1.2	C_15_H_18_O_8_	2
**8**	4-*O*-caffeoylquinic acid	5.7	353.0873	353.0869	-1.1	C_16_H_18_O_9_	2
**9**	apigenin 6,8-di-*C*-hexoside	7.9	593.1506	593.1497	-1.5	C_27_H_30_O_15_	2
**10**	3-*O*-feruloylquinic acid	8.2	367.1029	367.1024	-1.4	C_17_H_20_O_9_	2
**11**	methyl chlorogenate	8.7	367.1029	367.1021	-2.2	C_17_H_20_O_9_	2
**12**	scopoletin ^a^	9.2	191.0344	191.0340	-2.1	C_10_H_8_O_4_	1
**13**	feruloyl hexose	9.4	355.1029	355.1015	-3.9	C_16_H_20_O_9_	2
**14**	quercetin 3-*O*-neohesperidoside	9.6	609.1456	609.1470	2.3	C_27_H_30_O_6_	2
**15**	monohydroxy-dimethoxycoumarin	9.8	221.0450	221.0442	-2.3	C_11_H_10_O_5_	3
**16**	hyperoside	9.8	463.0877	463.0875	-0.4	C_21_H_20_O_12_	1
**17**	isoquercitrin	10.0	463.0877	463.0874	-0.6	C_21_H_20_O_12_	2
**18**	luteolin 7-*O*-glucoside	10.3	447.0927	447.0932	1.1	C_21_H_20_O_11_	1
**19**	methoxy-pentahydroxy(iso)flavone-*O*-hexoside	10.4	493.0982	493.0981	-0.2	C_22_H_22_O_13_	3
**20**	quercetin 3-*O*-acetylhexoside	10.8	505.0982	505.0982	0.0	C_23_H_22_O_13_	2
**21**	kaempferol-3-*O*-rutinoside	11.0	593.1506	593.1509	0.5	C_27_H_30_O_15_	2
**22**	3,4-*O*-dicaffeoylquinic acid	11.2	515.1190	515.1202	2.3	C_25_H_24_O_12_	2
**23**	1,5-*O*-dicaffeoylquinic acid	11.4	515.1190	515.1202	2.3	C_25_H_24_O_12_	3
**24**	3,5-*O*-dicaffeoylquinic acid	11.5	515.1190	515.1205	2.9	C_25_H_24_O_12_	2
**25**	isorhamnetin 3-*O*-*β*-d-glucoside	12.1	477.1030	477.1028	0.4	C_22_H_22_O_12_	1
**26**	quercetin 3-*O*-acetylhexoside	12.3	505.0982	505.0983	0.2	C_23_H_22_O_13_	2
**27**	4,5-*O*-dicaffeoylquinic acid	13.0	515.1190	515.1184	-1.2	C_25_H_24_O_12_	2
**28**	monohydroxy-dimethoxycoumarin	14.2	221.0450	221.0443	-3.2	C_11_H_10_O_5_	2

**Fig 6:**
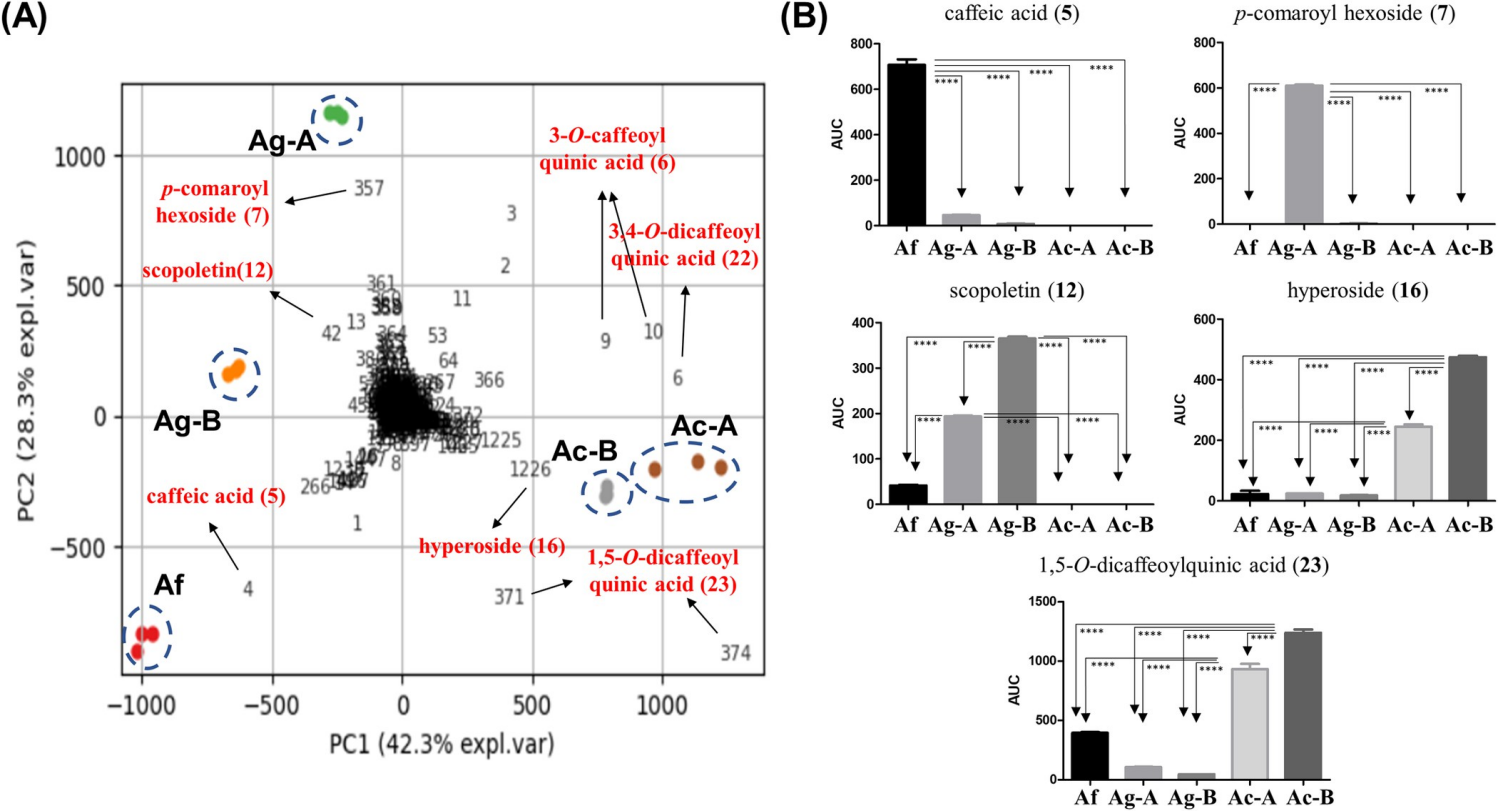
(A) The PCA biplot of the *Artemisia* samples represented by group marks (Af: *A*. *fukudo*; Ag-A: *A*. *gmelinii*-A; Ag-B: *A*. *gmelinii*-B; Ac-A: *A*. *capillaris*-A; Ac-B: *A*. *capillaris*-B). The numbers denote PCA loadings of each MS feature. Important marker MS features are labeled with their annotation and peak numbers enlisted in Table 3. Most MS features shown here are [M−H]^−^ ions, but MS features 9 and 372 are in-source fragments of peaks **6** and **23**, respectively. (B) Bar plots showing the ion intensities of selected marker peaks (**5**, **7**, **12**, **16,** and **23**) in analyzed *Artemisia* samples (**** *p* < 0.0001).

For the comparative analysis between the specialized metabolome of three *Artemisia* species, we extracted and normalized 1,763 MS features from the LC-MS data and applied them to principal component analysis (PCA). After technical validation with QC samples, the PCA biplot was visualized to reveal the chemical markers showing the most significantly different content between samples. The PC1 score, accounting for 42.3% of the total variance, discriminated *A*. *capillaris* from the other two species, while the PC2 score discriminated *A*. *gmelinii* and *A*. *fukudo*, accounting for 28.3% of the total variance (Fig 6A). PCA loading vectors revealed which MS features show relatively large ion intensity in each species. Caffeic acid (**5**, marked as MS feature 4 in Fig 6A) was abundant in *A*. *fukudo*, while *p*-coumaric acid hexoside (**7**, MS feature 357) and scopoletin (**12**, MS feature 42) showed relatively high contents in *A*. *gmelinii*. Multiple compounds such as 3-*O*-caffeoylquinic acid (**6**, MS features 9 and 10), 3,4-*O*-dicaffeoylquinic acid (**22**, MS feature 6), 1,5-*O*-dicaffeoylquinic acid (**23**, MS features 371 and 374), and hyperoside (**16**, MS feature 1226) showed relatively high ion abundances in the two *A*. *capillaris* samples compared to the others.

The ion abundances of MS features suggested as discriminating variables in PCA (peaks **5**, **6**, **7**, **12**, **16**, **22**, and **23**) were further compared as shown in Fig 6B. Peak **5** showed significantly higher contents in *A*. *fukudo* than in the others, which supported **5** as a potential chemical marker for *A*. *fukudo*. Peak **16** also showed species-specific occurrence in *A*. *capillaris*, although the relative abundance of **16** showed 2-fold differences between biological replicates. Peak **12** were comparatively abundant in *A*. *gmelinii* while **23** showed high concentration in *A*. *fukudo*; however, *A*. *fukudo* also contained both compounds, so further data should be acquired from more individuals to validate if this trend is general. In contrast, peaks **6** and **22** were ubiquitous in the three species, and their interspecific variations were not significantly larger than the intraspecific variations within *A*. *gmelinii* and *A*. *capillaris* (S4 Fig).

Peak **7** highlighted the reason why a large population of sample should be considered for developing chemical markers. Despite its abundance in *A*. *gmelinii*-A, it showed only a trace amount in *A*. *gmelinii*-B. This result suggests that the intraspecific genetic variation can cause about 100-fold difference on a level of a metabolite, as we controlled the environmental factors by growing these samples within a same greenhouse. Thus, more data from samples acquired from larger genetic pools, in addition to larger environmental and geographical varieties, should be inspected to develop valid chemical markers for *Artemisia* species.

## Conclusion

This study identified molecular markers that can be used for the authentication of three medicinal plants similar in appearance: *A*. *capillaris*, *A*. *gmelinii*, and *A*. *fukudo*. Comparative analysis of the complete chloroplast genomes of these plants suggested seven InDel-based DNA barcodes, and these markers successfully identified *Artemisia* species in the market. In addition, we analyzed specialized metabolites of the *Artemisia* species grown in a same environment using LC-MS/MS. It revealed multiple metabolites showing significant interspecific variation. These metabolites can be potential chemical markers, although further intensive efforts are required to validate those as chemical markers. By integrating DNA barcoding and chemical profiling, these species can be identified, authenticated, and monitored throughout the production process of botanical goods i.e., cultivation, harvest, circulation, and extraction.

## Supporting information

S1 Fig Sequence variation in the developed DNA barcode markers between species. AG, *A*. *gmelinii*; AC, *A*. *capillaris*; AF, *A*. *fukudo*.
(PDF)

S2 Fig The result of the blind test on commercial *Artemisia* samples, using five InDel-based barcode markers for identification. (PDF)

S3 Fig The mirror plot of experimental MS/MS spectra (upper, black) and GNPS reference spectra (lower, green) for the metabolite annotation. (PDF)

S4 Fig Bar plots showing the ion intensities of peaks 6 and 22 in analyzed *Artemisia* samples. (PDF)

S1 Table Detailed information of the plant materials used in this study. (PDF)

S2 Table Accession numbers of the chloroplast genomes used in this study. (PDF)

S3 Table Primer sets used in this study. (PDF)

S4 Table Detailed parameters used for the feature finding in MZmine 2. (PDF)

S1 File Detailed MS acquisition parameters. (PDF)

S1 Raw images Raw gel images. (PDF)
