# Correction: Genetic and chemical markers for authentication of three *Artemisia* species: *A*. *capillaris*, *A*. *gmelinii*, and *A*. *fukudo*

**DOI:** 10.1371/journal.pone.0293767

**Published:** 2023-10-26

**Authors:** Yun Sun Lee, Sunmin Woo, Jin-Kyung Kim, Jee Young Park, Nur Kholilatul Izzah, Hyun-Seung Park, Jung Hwa Kang, Taek Joo Lee, Sang Hyun Sung, Kyo Bin Kang, Tae-Jin Yang

There is an error in affiliation 1 for authors Yun Sun Lee, Jin-Kyung Kim, Jee Young Park, Nur Kholilatul Izzah, Hyun-Seung Park, and Tae-Jin Yang. The correct affiliation 1 is: Department of Agriculture, Forestry and Bioresources, Plant Genomics and Breeding Institute, College of Agriculture and Life Sciences, Seoul National University, Seoul, Republic of Korea.

## References

[1] Lee YS, Woo S, Kim J-K, Park JY, Izzah NK, Park H-S, et al. (2022) Genetic and chemical markers for authentication of three *Artemisia* species: *A*. *capillaris*, *A*. *gmelinii*, and *A*. *fukudo*. PLoS ONE 17(3): e0264576. 10.1371/journal.pone.0264576 35271607 PMC8912906

