# Supplementary material for: Genetic and chemical markers for authentication of three Artemisia species: A. capillaris, A. gmelinii, and A. fukudo
Source: PLoS One. 2022 Mar 10;17(3):e0264576. doi: 10.1371/journal.pone.0264576 (PMC8912906; doi:10.1371/journal.pone.0264576)
Supplement: S1 Fig — AG, A. gmelinii; AC, A. capillaris; AF, A. fukudo. (PDF) [file pone.0264576.s001.pdf]

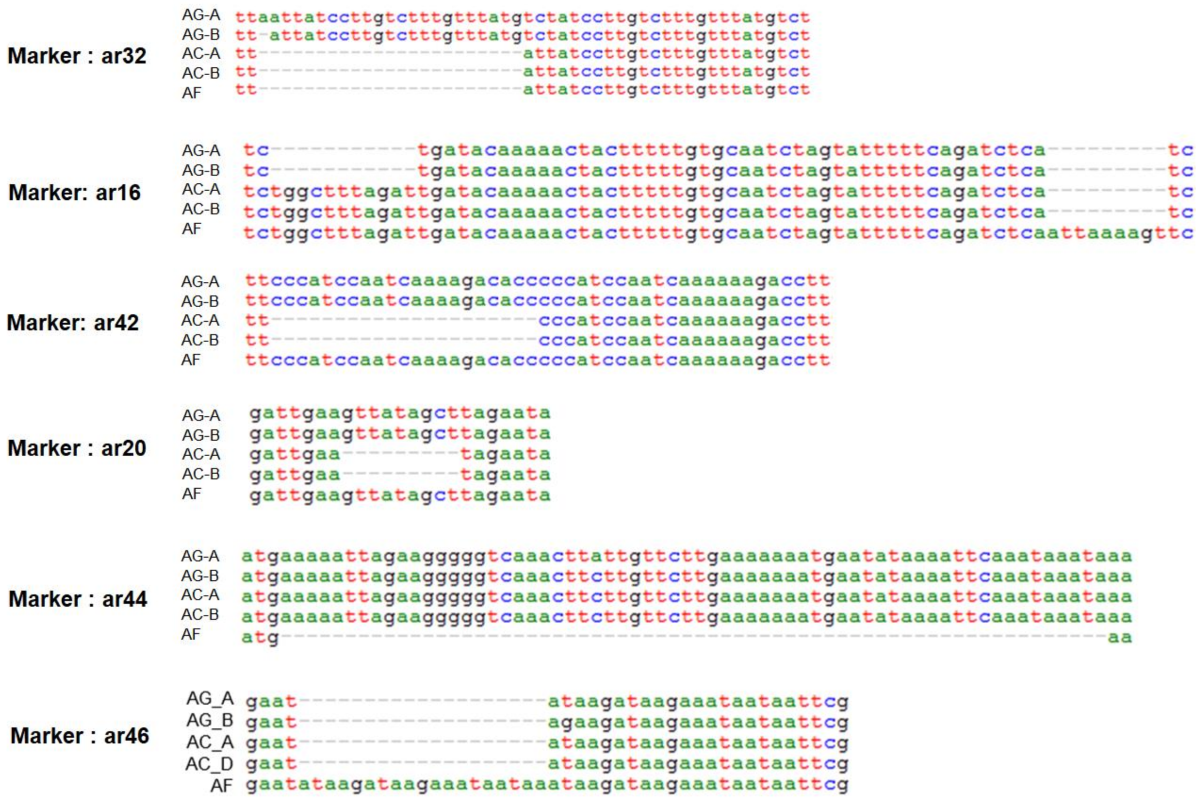

S1 Fig. Sequence variation in developed DNA barcode markers between the species. AG, *A. gmelinii*; AC, *A. capillaris*; AF, *A. fukudo*
