# Supplementary material for: Genetic and chemical markers for authentication of three Artemisia species: A. capillaris, A. gmelinii, and A. fukudo
Source: PLoS One. 2022 Mar 10;17(3):e0264576. doi: 10.1371/journal.pone.0264576 (PMC8912906; doi:10.1371/journal.pone.0264576)
Supplement: S2 Fig — (PDF) [file pone.0264576.s002.pdf]

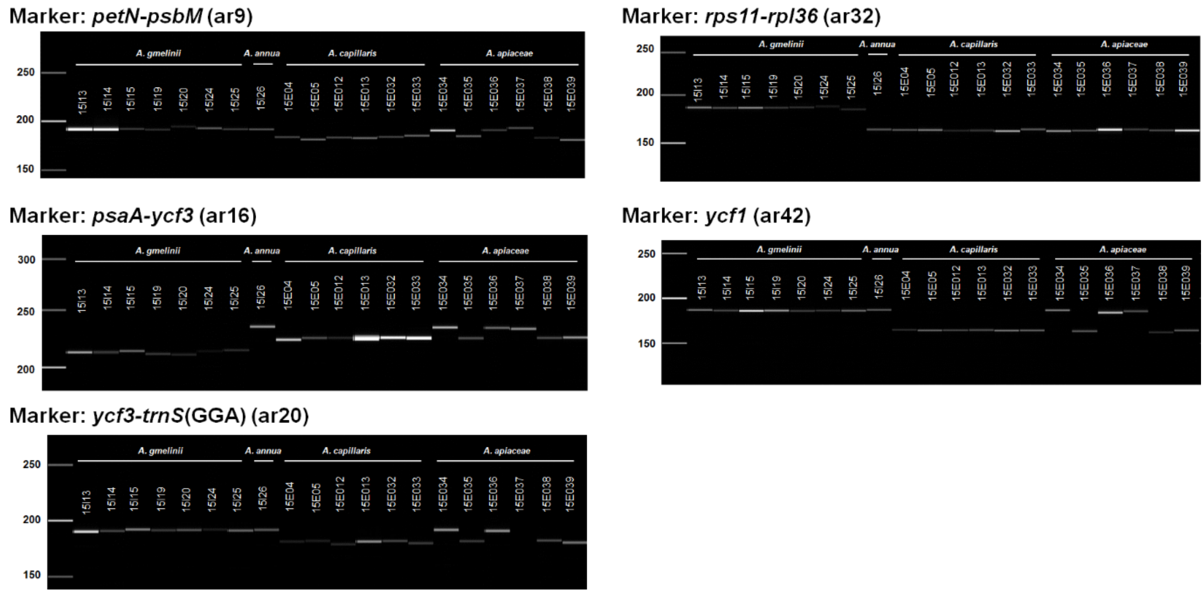

S2 Fig. The result of the blind test on commercial *Artemisia* samples identified using five InDel-based barcode markers.
