# Supplementary material for: Genetic and chemical markers for authentication of three Artemisia species: A. capillaris, A. gmelinii, and A. fukudo
Source: PLoS One. 2022 Mar 10;17(3):e0264576. doi: 10.1371/journal.pone.0264576 (PMC8912906; doi:10.1371/journal.pone.0264576)
Supplement: S3 Fig — (PDF) [file pone.0264576.s003.pdf]

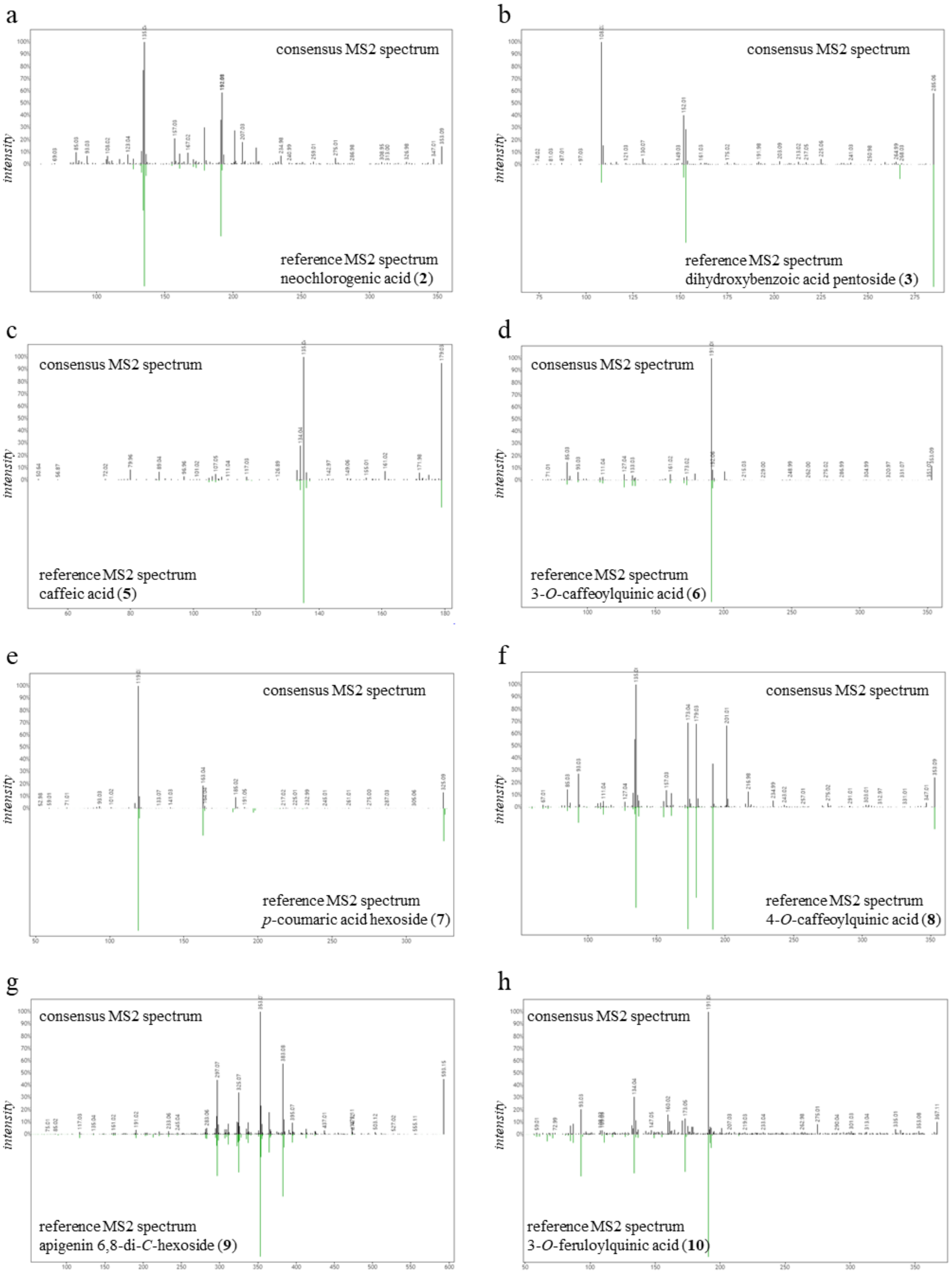
a
consensus MS2 spectrum
reference MS2 spectrum
neochlorogenic acid (**2**)
intensity
b
consensus MS2 spectrum
reference MS2 spectrum
dihydroxybenzoic acid pentoside (**3**)
intensity
c
consensus MS2 spectrum
reference MS2 spectrum
caffeic acid (**5**)
intensity
d
consensus MS2 spectrum
reference MS2 spectrum
3-*O*-caffeoylquinic acid (**6**)
intensity
e
consensus MS2 spectrum
reference MS2 spectrum
p-coumaric acid hexoside (**7**)
intensity
f
consensus MS2 spectrum
reference MS2 spectrum
4-*O*-caffeoylquinic acid (**8**)
intensity
g
consensus MS2 spectrum
reference MS2 spectrum
apigenin 6,8-di-*C*-hexoside (**9**)
intensity
h
consensus MS2 spectrum
reference MS2 spectrum
3-*O*-feruloylquinic acid (**10**)
intensity

(continued)

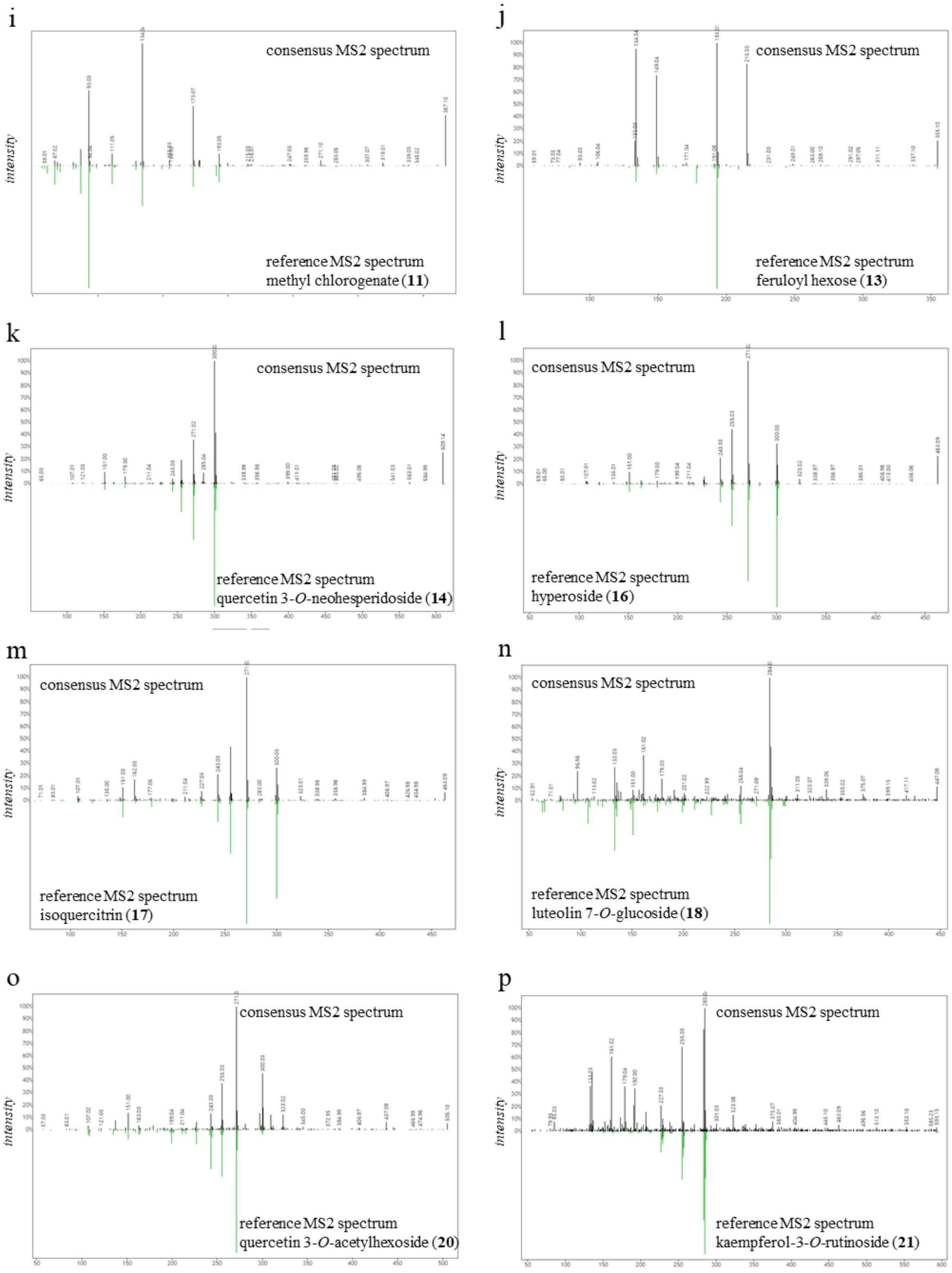
i
consensus MS2 spectrum
intensity
reference MS2 spectrum
methyl chlorogenate (11)
j
consensus MS2 spectrum
intensity
reference MS2 spectrum
feruloyl hexose (13)
k
consensus MS2 spectrum
intensity
reference MS2 spectrum
quercetin 3-O-neohesperidoside (14)
l
consensus MS2 spectrum
intensity
reference MS2 spectrum
hyperoside (16)
m
consensus MS2 spectrum
intensity
reference MS2 spectrum
isoquercitrin (17)
n
consensus MS2 spectrum
intensity
reference MS2 spectrum
luteolin 7-O-glucoside (18)
o
consensus MS2 spectrum
intensity
reference MS2 spectrum
quercetin 3-O-acetylhexoside (20)
p
consensus MS2 spectrum
intensity
reference MS2 spectrum
kaempferol-3-O-rutinoside (21)

(continued)

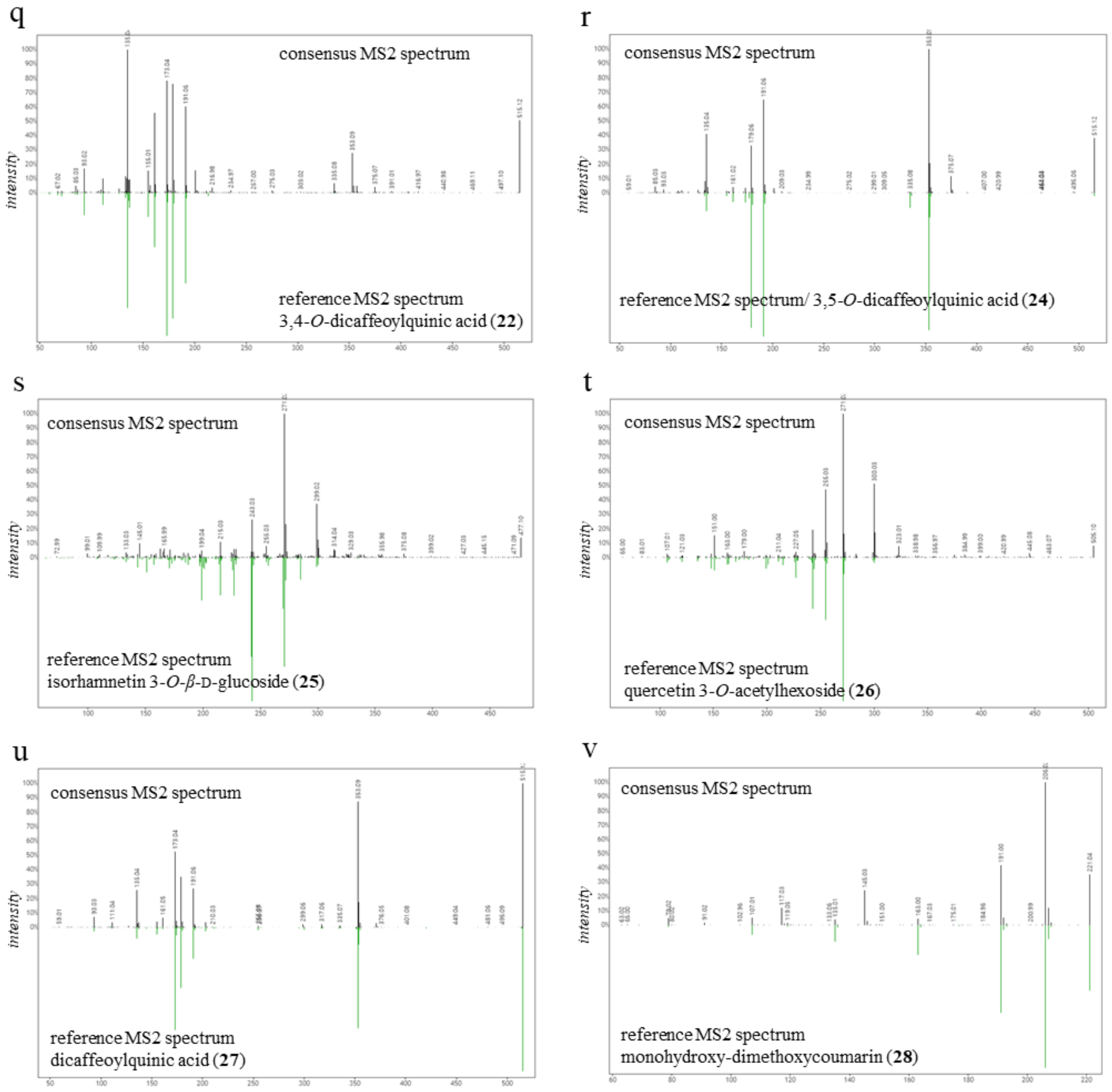

S3 Fig. The mirror plot of experimental MS/MS spectra (upper, black) and GNPS reference spectra (lower, green) for the metabolite annotation.
