# Supplementary figures and images for: Genetic and chemical markers for authentication of three Artemisia species: A. capillaris, A. gmelinii, and A. fukudo
Source: PLoS One. 2022 Mar 10;17(3):e0264576. doi: 10.1371/journal.pone.0264576 (PMC8912906; doi:10.1371/journal.pone.0264576)

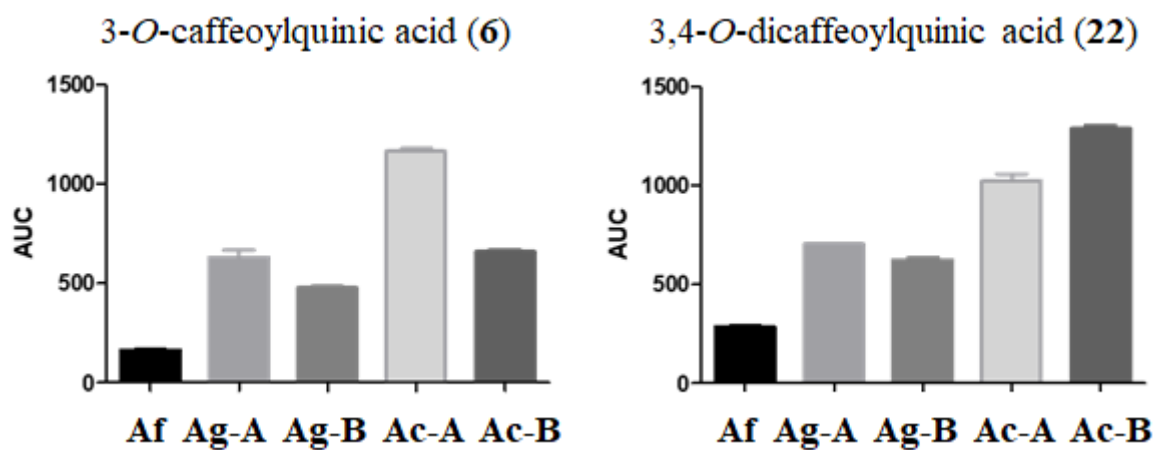

S4 Fig. Bar plots showing the ion intensities of peaks 6 and 22 in analyzed *Artemisia* samples.

Supplement: S4 Fig — (PDF) [file pone.0264576.s004.pdf]
