# Supplementary material for: Genetic and chemical markers for authentication of three Artemisia species: A. capillaris, A. gmelinii, and A. fukudo
Source: PLoS One. 2022 Mar 10;17(3):e0264576. doi: 10.1371/journal.pone.0264576 (PMC8912906; doi:10.1371/journal.pone.0264576)
Supplement: S1 Table — (PDF) [file pone.0264576.s005.pdf]

S1 Table. Detailed information of the plant materials used in this study.

species	collected site	maintenance site
A. gmelinii-A	Hongcheon, Korea (37° 41' N, 127° 53' E)	Hantaek Botanical Garden, Yongin, Korea
A. gmelinii-B	Pyeongchang, Korea (37° 25'N, 128° 31' E)	Department of Herbal Crop Research, Rural Development Administration, Eumseong, Korea
A. capillaris-A	Taean, Korea (36°50'N 126°11'E)	Hantaek Botanical Garden, Yongin, Korea
A. capillaris-B	Eumseong, Korea (36°58'N, 127°36'E)	Department of Herbal Crop Research, Rural Development Administration, Eumseong, Korea
A. fukudo	Jeungdo, Korea (34°59'N 126°10'E)	Hantaek Botanical Garden, Yongin, Korea
