# Supplementary material for: Genetic and chemical markers for authentication of three Artemisia species: A. capillaris, A. gmelinii, and A. fukudo
Source: PLoS One. 2022 Mar 10;17(3):e0264576. doi: 10.1371/journal.pone.0264576 (PMC8912906; doi:10.1371/journal.pone.0264576)
Supplement: S2 Table — (PDF) [file pone.0264576.s006.pdf]

S2 Table. Accession numbers of the chloroplast genomes used in this study.

subfamily	tribe	species	GenBank accession number
Asteroideae	Anthemideae	*Artemisia fukudo*	KU360270
		Artemisia frigida	JX293720
		Artemisia montana	KF887960
		Artemisia argyi	NC030785.1
		Artemisia gmelinii-A	KU736962
		Artemisia gmelinii-B	KU736963
		Artemisia capillaris-A	KU736963
		Artemisia capillaris-B	KU360270
	Anthemideae	*Chrysanthemum* × *morifolium*	JQ362483
	Astereae	*Aster spathulifolius*	KF279514
	Gnaphalieae	*Leontopodium leiolepis*	NC_027835.1
	Senecioneae	*Jacobaea vulgaris*	NC_015543.1
	Heliantheae alliance	*Helianthus annuus*	DQ383815
Cichorioideae	Cichorieae	*Guizotia abyssinica*	EU549769.1
		Lactuca sativa	AP007232.1
		Taraxacum officinale	KU361241.1
Carduoideae	Cardueae	*Silybum marianum*	NC_028027.1
		Cynara cardunculus var. *scolymus*	KP842709.1
