# Supplementary material for: Genetic and chemical markers for authentication of three Artemisia species: A. capillaris, A. gmelinii, and A. fukudo
Source: PLoS One. 2022 Mar 10;17(3):e0264576. doi: 10.1371/journal.pone.0264576 (PMC8912906; doi:10.1371/journal.pone.0264576)
Supplement: S3 Table — (PDF) [file pone.0264576.s007.pdf]

S3 Table. Primer sets used in this study.

marker name	locus	direction	primer sequence (5′ → 3′)
ar09	*petN-psbM*	F	CGCCGATTATAGCCCCTTGA
		R	TGCATTTATTGCTACTGCACTGT
ar16	*psaA-ycf3*	F	GATCTGGAAGTCGATCCGGG
		R	TAATGCCTGCTCGCGTGATT
ar20	*ycf3-trnS(GGA)*	F	GGGAACCGGATTCCATTCCAT
		R	GCGTAGTGTATACCCCTACAACA
ar32	*rpl36-infA*	F	ACGTCCACGTGAACCCTTTT
		R	ACGTCGTAGGGGACGAATTA
ar42	*ycf1*	F	CTTTCGGATTCGATATGAGGTGA
		R	ACCTAATAAGGACCAAATAACGGA
ar44	*lhbA-trnG(UCC)*	F	AGGTACATCGTTATGGCTTGGA
		R	ATATTTGTGCAGTGCTGGGC
ar46	*ndhF-rpl32*	F	TCCCATAGCAGATGAGATATAAACA
		R	AGTTGGATGTGAAAGACATCTGTTG
