# Supplementary material for: Genetic and chemical markers for authentication of three Artemisia species: A. capillaris, A. gmelinii, and A. fukudo
Source: PLoS One. 2022 Mar 10;17(3):e0264576. doi: 10.1371/journal.pone.0264576 (PMC8912906; doi:10.1371/journal.pone.0264576)
Supplement: S4 Table — (PDF) [file pone.0264576.s008.pdf]

S4 Table. Detailed parameters used for the feature finding in MZmine 2.

processing module	parameters	
crop filter	t_R range	2.00–20.00 min
peak detection	noise level for MS1	1.0E2
	noise level for MS2	0.0
chromatogram builder	min time span	0.25
	min highest intensity	1.0E3
	m/z tolerance	0.005 Da or 30 ppm
chromatogram deconvolution (local minimum search)	chromatographic threshold	80%
	min relative peak height	0.05
	search minimum in t_R range	0.05
	min absolute height	1.0E3
isotopic peak grouper	*m/z* tolerance	0.005 Da or 20 ppm
	t_R tolerance	0.5 min
	max charge	3
alignment (join aligner)	t_R tolerance (50 % weighted)	0.25 min
	m/z tolerance (50 % weighted)	0.005 Da or 20 ppm
gap-filling	intensity tolerance	10 %
	t_R tolerance	0.25 min
	m/z tolerance	0.005 Da or 20 ppm
normalization	to the peak area of IS (chicoric acid)	*m/z* 473–474<br>t_R 8.50–9.50 min
