# Supplementary material for: Genetic and chemical markers for authentication of three Artemisia species: A. capillaris, A. gmelinii, and A. fukudo
Source: PLoS One. 2022 Mar 10;17(3):e0264576. doi: 10.1371/journal.pone.0264576 (PMC8912906; doi:10.1371/journal.pone.0264576)
Supplement: S1 File — (PDF) [file pone.0264576.s009.pdf]

S1 File. Detailed MS acquisition parameters

MS detection was performed with an ESI interface in negative ion mode. Full scan mass spectra were recorded in centroid mode through a range of *m/z* 50–1,500 with scan duration of 0.2 sec for both of low- and high-energy scans. The $[M-H]^-$ ion of leucine enkephalin at *m/z* 554.2615 was used as the lockmass to ensure mass accuracy and reproducibility. Nitrogen was used as the nebulizer and argon was utilized as the collision gas. The ESI source was operated with a capillary voltage of 3 kV, sampling cone voltage of 45 V, extraction cone voltage of 4 V, cone gas flow of 50 L/h, desolvation gas flow of 800 L/h, source temperature of 120°C, desolvation temperature of 350°C, and CID energy ramped from 40 to 45 eV. Data acquisition and processing were performed with the software MassLynx™ (Waters Corporation, Manchester, UK).
