# Supplementary material for: Genetic and chemical markers for authentication of three Artemisia species: A. capillaris, A. gmelinii, and A. fukudo
Source: PLoS One. 2022 Mar 10;17(3):e0264576. doi: 10.1371/journal.pone.0264576 (PMC8912906; doi:10.1371/journal.pone.0264576)
Supplement: S1 Raw images — (PDF) [file pone.0264576.s010.pdf]

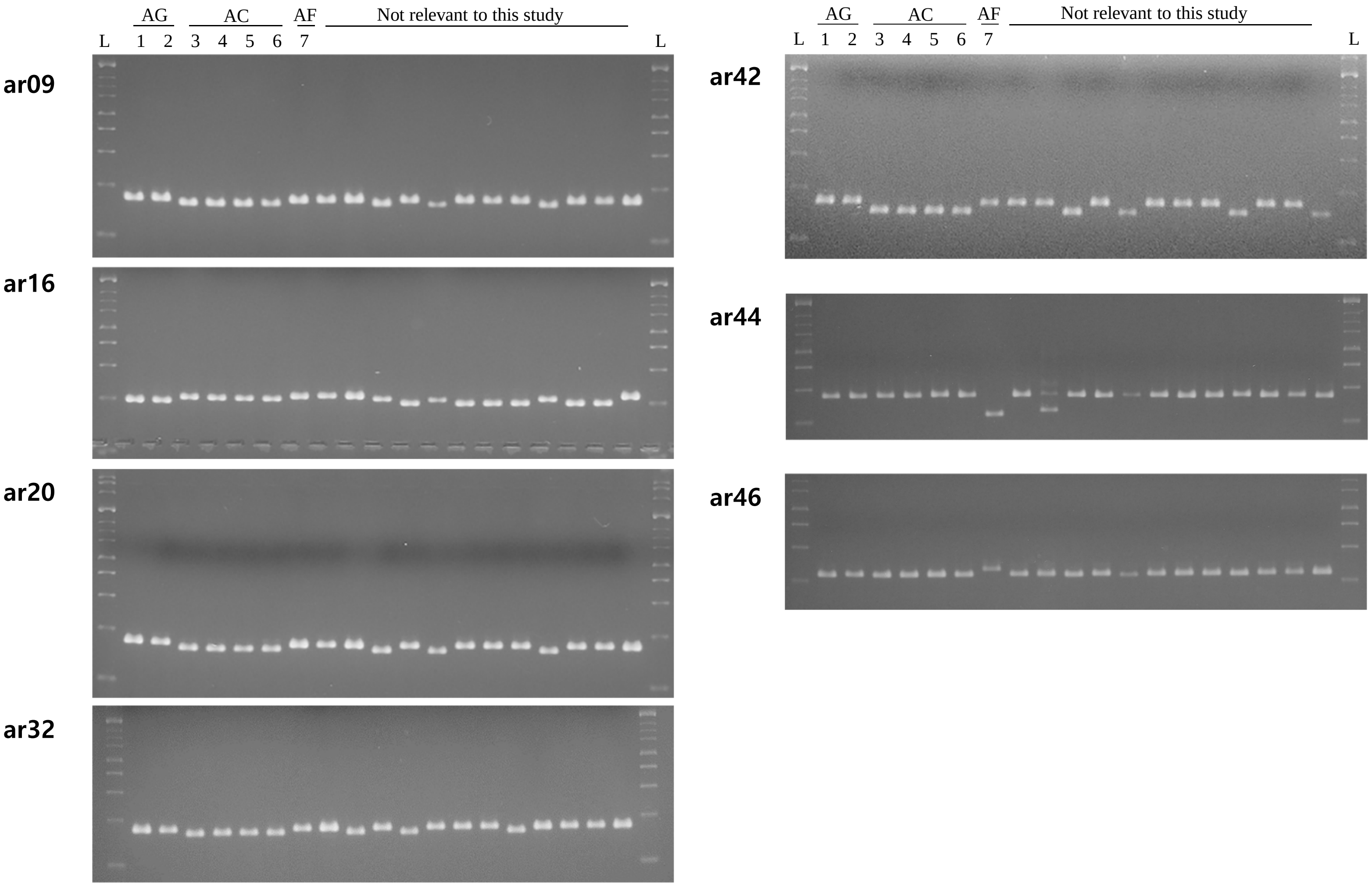

Raw gel image for Figure 4

Marker: ***petN-psbM*** **(ar9)**

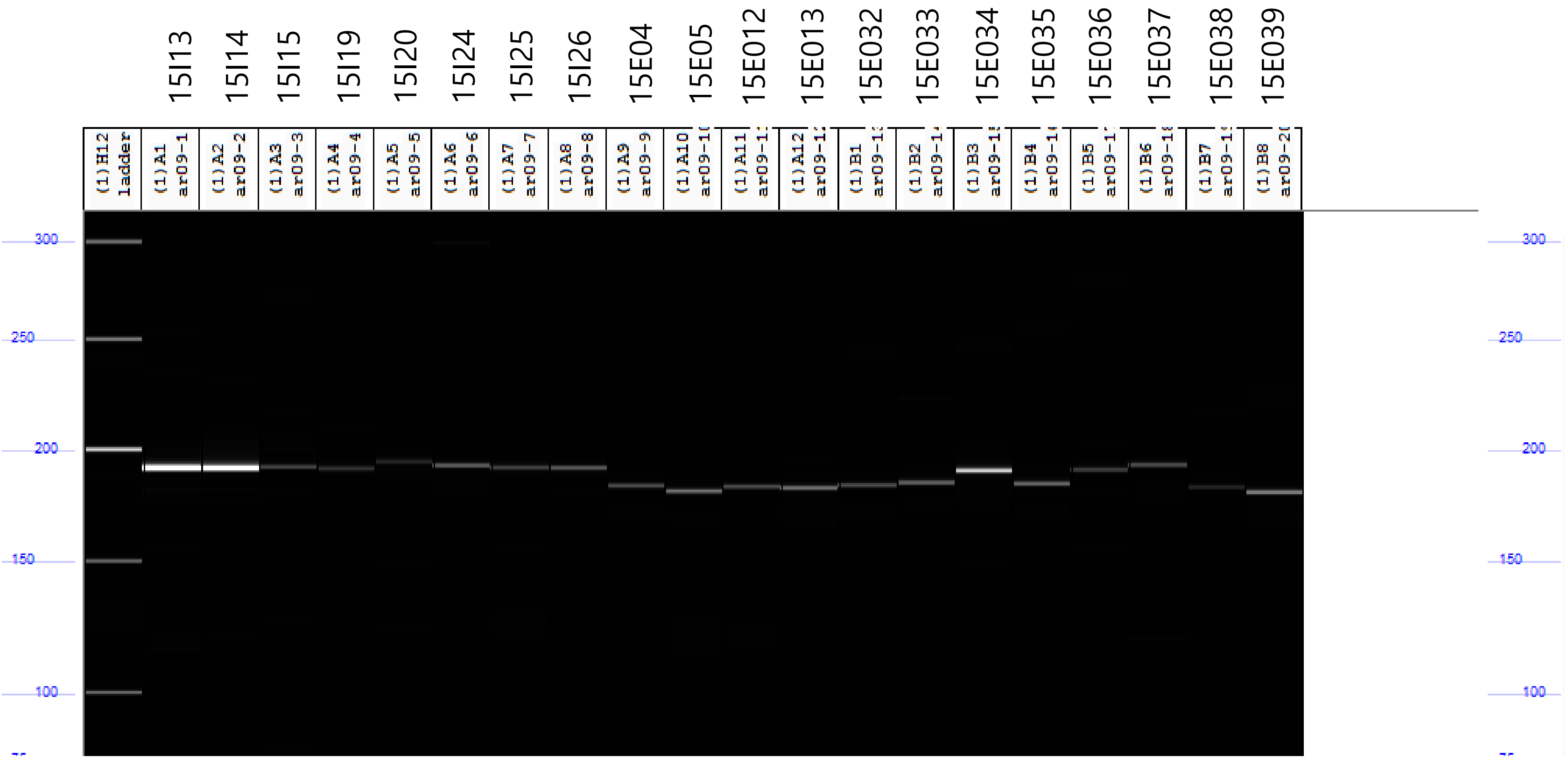

Raw gel image for Figure S2

Marker: *psaA-ycf3* (ar16)

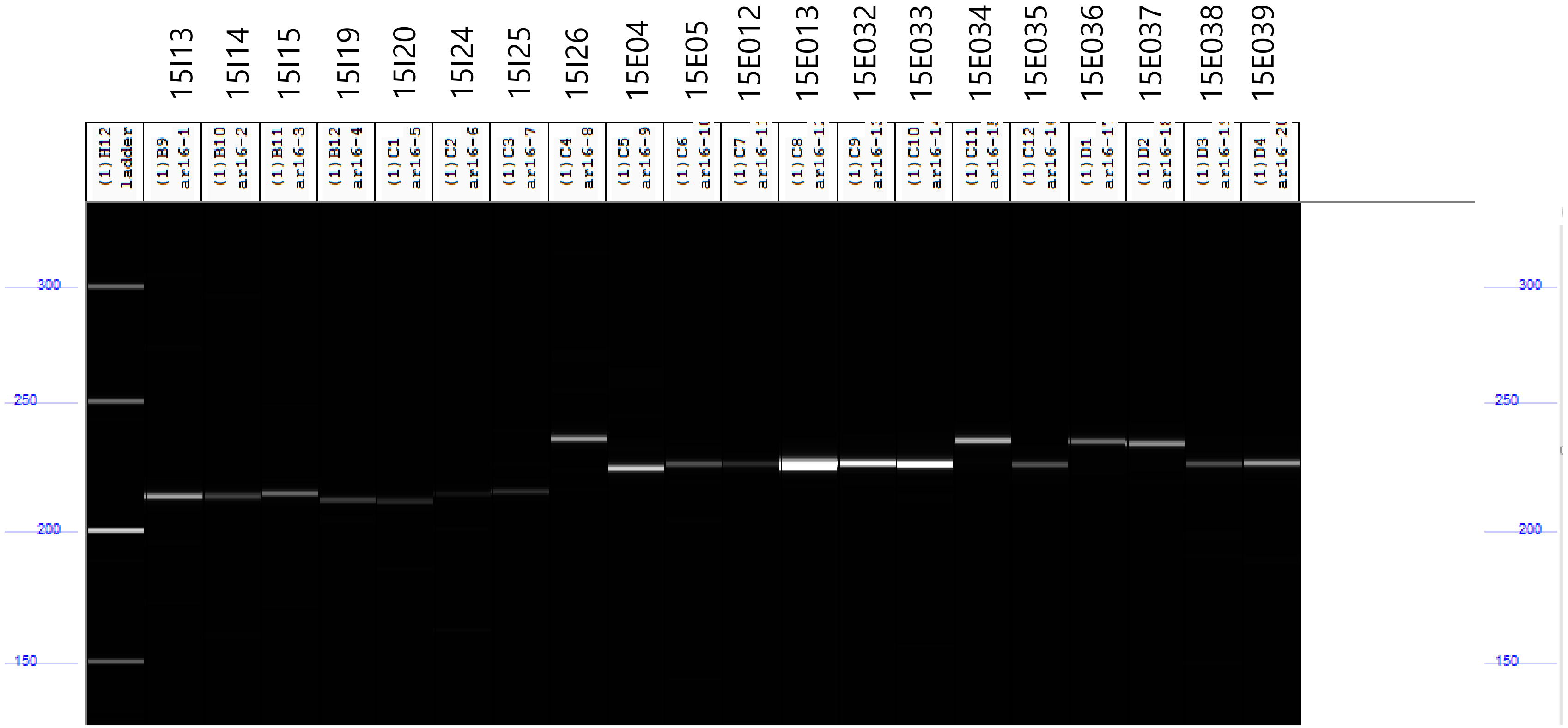

Raw gel image for Figure S2

Marker: *ycf3-trnS*(GGA) (ar20)

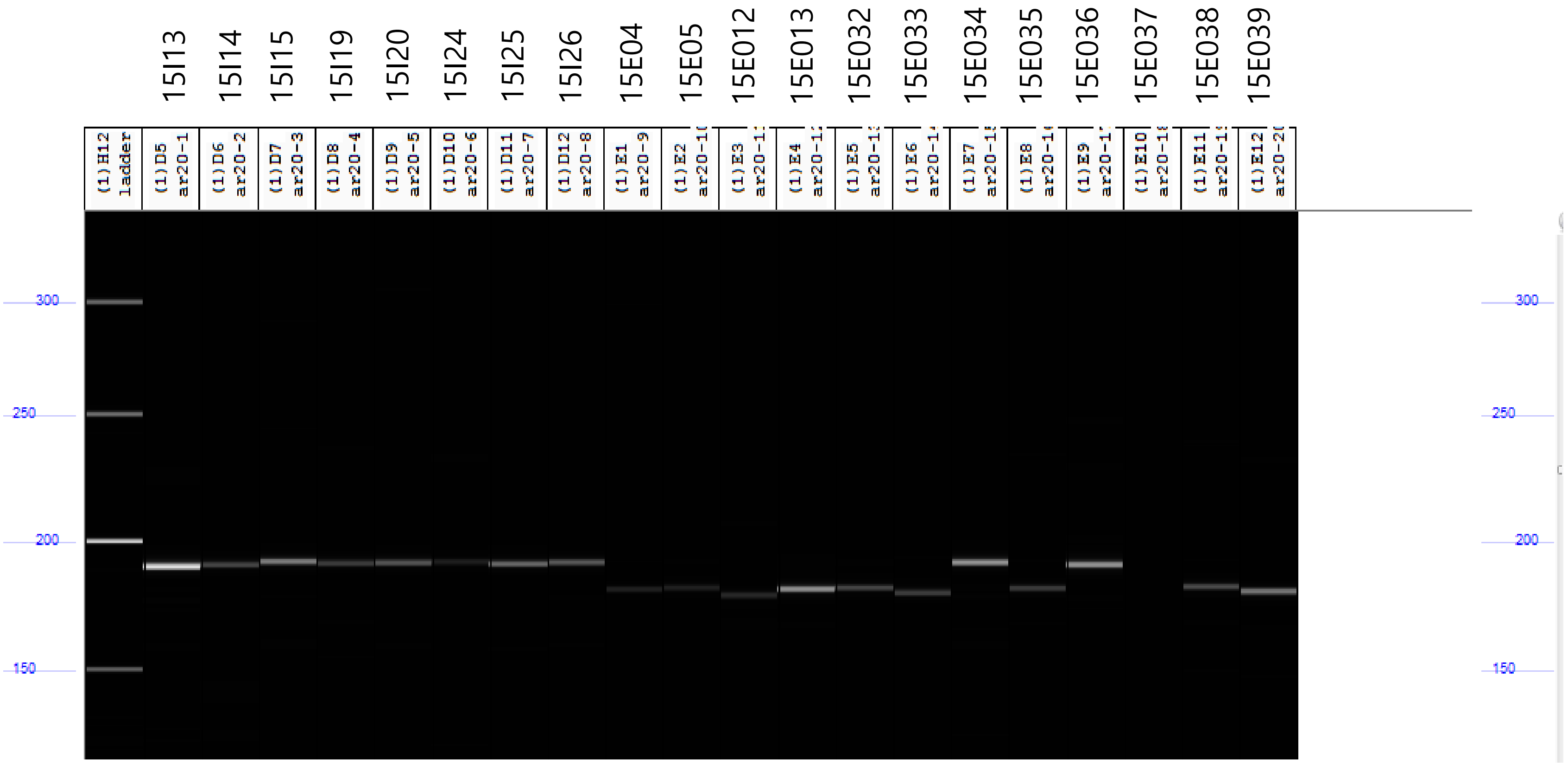

Raw gel image for Figure S2

Marker: ***rps11-rpl36*** **(ar32)**

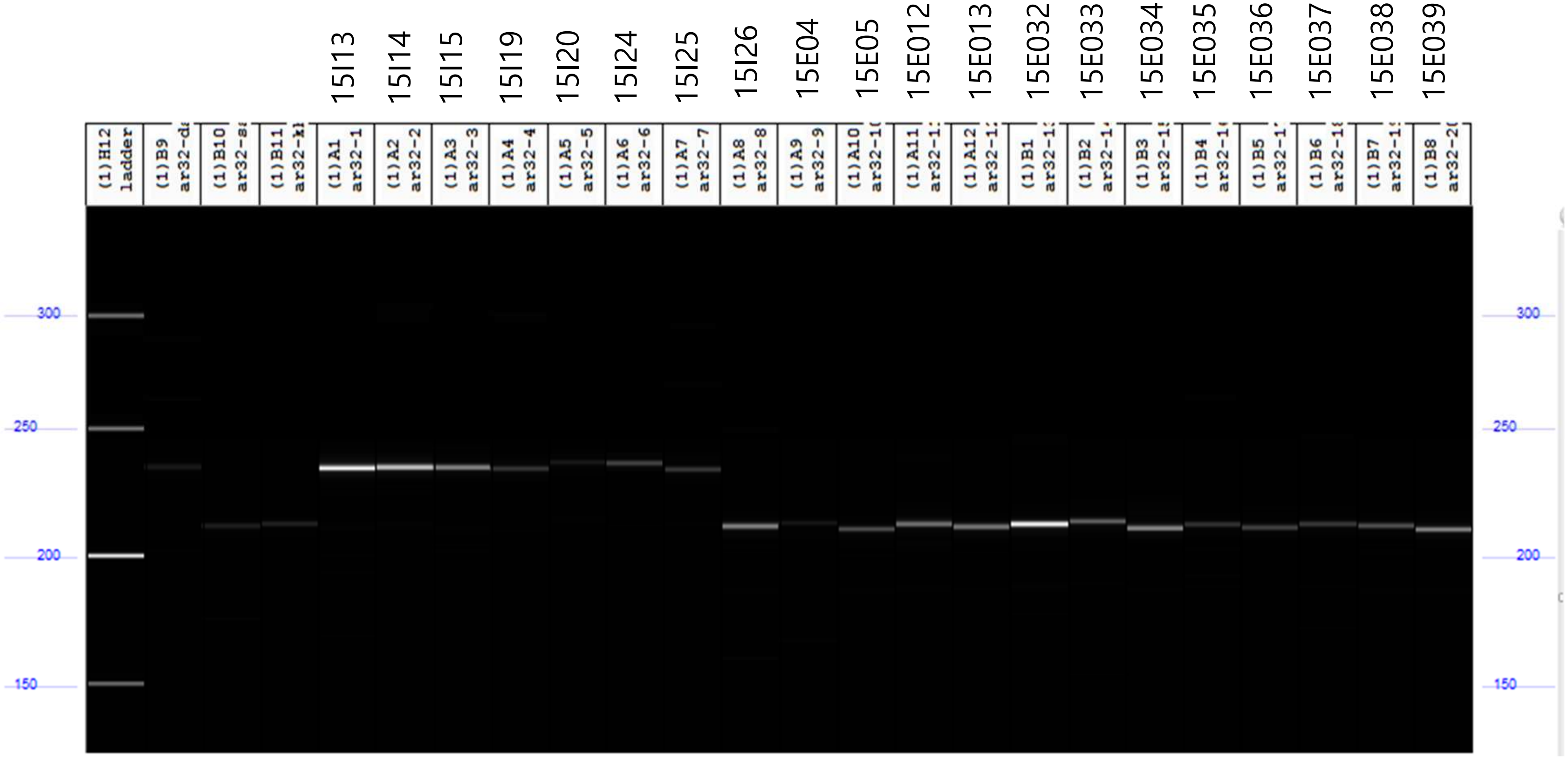

Raw gel image for Figure S2

Marker: *ycf1* (ar42)

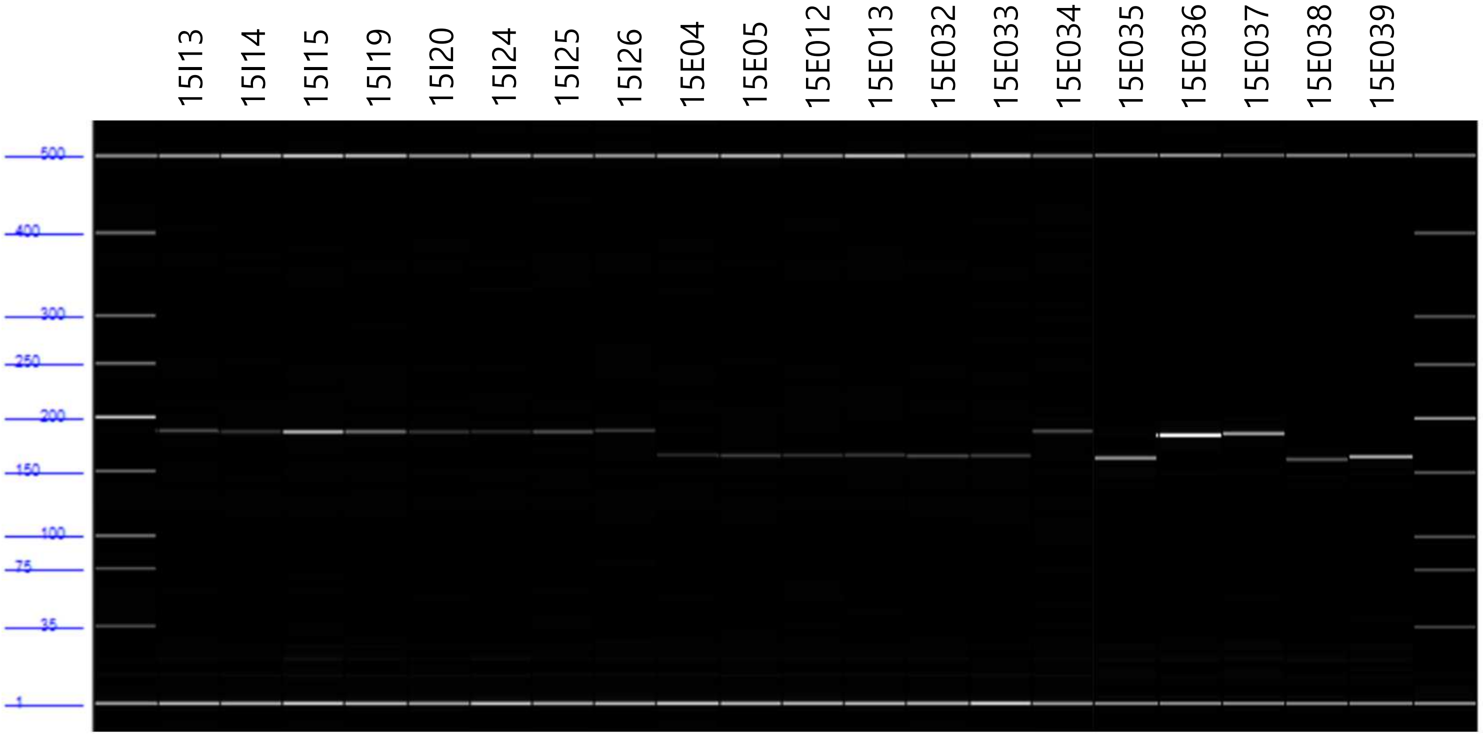

Raw gel image for Figure S2

< Notification >

- The gel blotting images provided here were detected by digital imaging, and the gels were not cut into smaller fragments or strips for processing.

- We tried to attach the original, uncropped images of gels; however, unfortunately we could not find the very original images, mainly because the images were taken in October 2015, which was about 6 years before submission of this manuscript. We guess the original image was lost while we replaced the PC used for the imaging system.

- The images provided here are the most unprocessed ones among the images available at this time. We are sure that only blank spaces without any PCR bands had been cropped from the original images.
